# Synthesis and Pharmacological Effects of Diosgenin–Betulinic Acid Conjugates

**DOI:** 10.3390/molecules25153546

**Published:** 2020-08-03

**Authors:** Zülal Özdemir, Michaela Rybková, Martin Vlk, David Šaman, Lucie Rárová, Zdeněk Wimmer

**Affiliations:** 1 Isotope Laboratory, Institute of Experimental Botany of the Czech Academy of Sciences, Vídeňská 1083, 14220 Prague 4, Czech Republic; zulalozdemr@gmail.com (Z.Ö.); martin.vlk@fjfi.cvut.cz (M.V.); 2 Department of Chemistry of Natural Compounds, University of Chemistry and Technology in Prague, Technická 5, 16628 Prague 6, Czech Republic; rybkova.misa@seznam.cz; 3 Faculty of Nuclear Sciences and Physical Engineering, Czech Technical University in Prague, Břehová 7, 11519 Prague 1, Czech Republic; 4 Department of NMR Spectroscopy, Institute of Organic Chemistry and Biochemistry of the Czech Academy of Sciences, Flemingovo náměstí 2, 16610 Prague 6, Czech Republic; nmrsaman@gmail.com; 5 Laboratory of Growth Regulators, Faculty of Science, Palacký University, and Institute of Experimental Botany of the Czech Academy of Sciences, Šlechtitelů 27, 78371 Olomouc, Czech Republic; lucie.rarova@upol.cz

**Keywords:** betulinic acid, diosgenin, Huisgen copper(I)-catalyzed 1,3-dipolar cycloaddition, catalytic hydrogenation, conjugate, adaptogen, cytotoxicity, ADME parameters

## Abstract

The target diosgenin–betulinic acid conjugates are reported to investigate their ability to enhance and modify the pharmacological effects of their components. The detailed synthetic procedure that includes copper(I)-catalyzed Huisgen 1,3-dipolar cycloaddition (click reaction), and palladium-catalyzed debenzylation by hydrogenolysis is described together with the results of cytotoxicity screening tests. Palladium-catalyzed debenzylation reaction of benzyl ester intermediates was the key step in this synthetic procedure due to the simultaneous presence of a 1,4-disubstituted 1,2,3-triazole ring in the molecule that was a competing coordination site for the palladium catalyst. High pressure (130 kPa) palladium-catalyzed procedure represented a successful synthetic step yielding the required products. The conjugate **7** showed selective cytotoxicity in human T-lymphoblastic leukemia (CEM) cancer cells (IC_50_ = 6.5 ± 1.1 µM), in contrast to the conjugate **8** showing no cytotoxicity, and diosgenin (**1**), an adaptogen, for which a potential to be active on central nervous system was calculated in silico. In addition, **5** showed medium multifarious cytotoxicity in human T-lymphoblastic leukemia (CEM), human cervical cancer (HeLa), and human colon cancer (HCT 116). Betulinic acid (**2**) and the intermediates **3** and **4** showed no cytotoxicity in the tested cancer cell lines. The experimental data obtained are supplemented by and compared with the in silico calculated physico-chemical and absorption, distribution, metabolism, and excretion (ADME) parameters of these compounds.

## 1. Introduction

Saponins belong among plant products displaying multiple biological and pharmacological activity, and many of them are considered to be adaptogens. This term is used to describe a plant product capable of (a) producing a nonspecific response resulting in increasing the power of resistance against multiple (physical, chemical, or biological) stressors; (b) having a normalizing effect, irrespective of the nature of the pathology, so thus being non-toxic; and (c) being harmless and not influencing normal body functions more than required [1].

**Figure 1:**
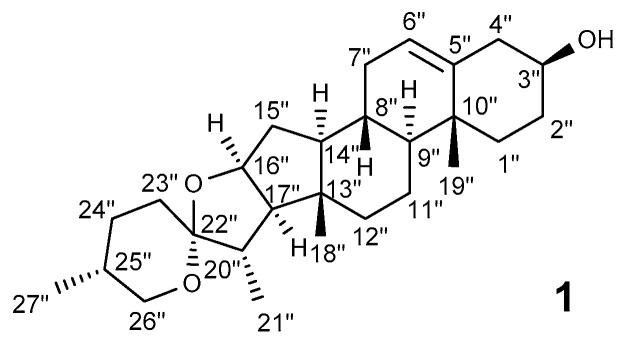
Structure of diosgenin (**1**). Note: carbon atoms are double-primed as in evaluation of the NMR spectra of diosgenin derivatives.

Diosgenin, (3β,25*R*)-spirost-5-en-3-ol (**1**, Figure 1), is a steroid sapogenin part of the saponin dioscin found in the tubers of *Dioscorea zingiberensis* C. H. Wright or *Trigonella foenum-graecum* L. and in numbers of legumes. Diosgenin is a widely used precursor in the synthesis of sexual hormones, peroral contraceptives and other steroids in the pharmaceutical industry [2]. It is an adaptogen, displaying non-steroidogenic activity along with other beneficial effects [3]. Diosgenin (**1**) is unable to bind metal ions, and therefore, the change made from more traditional cholesterol/cholesterylamine system to diosgenin could influence the overall conformation of the bivalent structures, modifying the metal ions chelating properties. Saponins are always species formed from an aglycone and several monosaccharide units, the presence of which increases the solubility of saponins in natural aqueous media [4].

During the past decade, a number of preclinical investigations have been made to elucidate chemopreventive and therapeutic effects of diosgenin [4]. In general, chemoprevention of cancer has been focused on a regression of multistage process of carcinogenesis. For that purpose, phytochemicals have been intensively applied due to their high safety, low toxicity, and almost no side effects (cf. definition for adaptogens above). So far, the main antitumor effect of diosgenin has been observed in treatment of colon cancer [5]. It affects cell growth and differentiation, and it also induces apoptosis in HT-29 human colon cancer cell line [5]. It induces caspase-3 expression and suppresses anti-apoptotic gene Bcl-2 [5]. Diosgenin is not metabolized in the human body, and it is considered to represent a safe natural drug. It has also been investigated for treating hyperglycemia, hypercholesterolemia, hypertriacylglycerolemia, and Alzheimer’s disease [4,6].

**Scheme 1:**
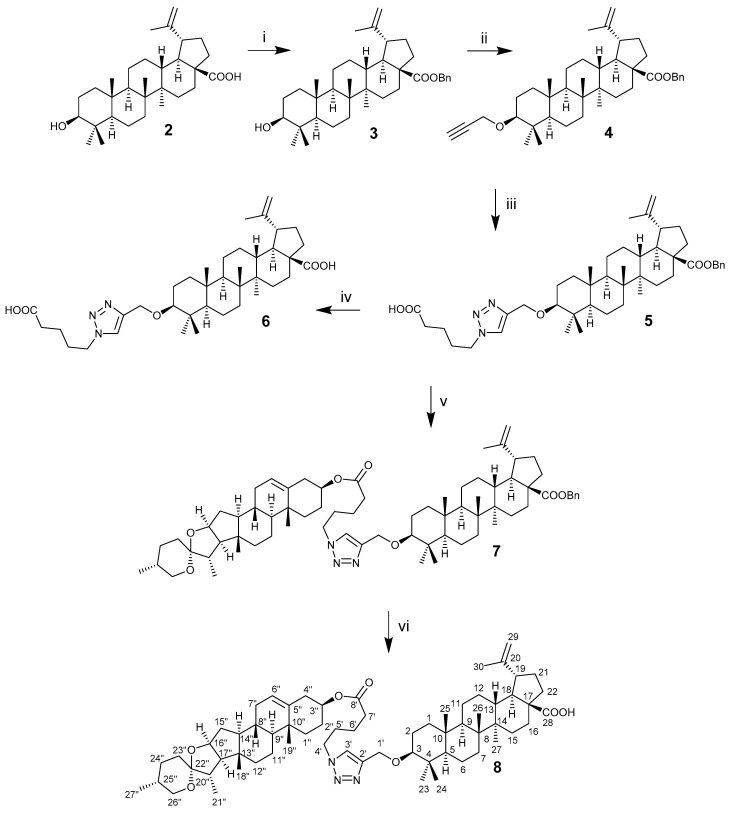
Synthetic procedure. Reagents used: i: benzyl bromide, K_2_CO_3_, DMF; ii: propargyl bromide, NaH, THF; iii: azidovaleric acid, CuSO_4_·5H_2_O, TBTA, sodium ascorbate, water/CH_2_Cl_2_ mixture; iv: H_2_ (Pd/C), 130 kPa, ethanol/THF mixture; v: **1**, DCC, CH_2_Cl_2_; vi: H_2_ (Pd/C), 130 kPa, ethanol/THF mixture.

Betulinic acid, 3β-hydroxylup-20(29)-en-28-oic acid (**2**; Scheme 1), is a pharmacologically perspective triterpenoid plant product with a broad spectrum of effects, e.g., antitumor, anti-HIV, cytostatic, and anti-inflammatory [7,8]. It can be obtained from the bark of *Betula pendula* Roth, widely distributed in Europe, and from a number of subtropical and tropical plants [7,8]. In 1995, a role of betulinic acid (**2**) in cell apoptosis was described [9].

The objectives of this investigation were: (a) to design simple conjugates on the basis of diosgenin (**1**) and betulinic acid (**2**) connected by a spacer bearing 1,4-disubstitued 1,2,3-triazole ring, (b) to investigate the key reaction steps of the synthetic procedure and to optimize them, as well as (c) to investigate the ability of such a kind of conjugate to enhance and/or to change the nature of the pharmacological effects of its components.

## 2. Results and Discussion

In this synthetic procedure (Scheme 1), several reaction steps were found requiring use of other approaches than originally planned ones. Either different reagents for successful completion of the target molecules had to be used or different approaches had to be selected. A selection of the reagents was often based on analogous synthetic procedures done earlier during our projects focused on steroids and triterpenoid acids [10]. Therefore, a more detailed investigation and/or optimization in the synthesis of **8** and **6** was made. We mostly focused on a behavior of the source molecules (**7** and **5**) during the synthesis, in which several procedures, earlier developed in our team, were used in the synthesis of triterpenoid acid derivatives, and in analogous reactions [10,11,12,13,14,15,16]. A hypothesis on the course of the palladium-catalyzed hydrogenation in its employed modifications is postulated.

The first step of the synthetic procedure (Scheme 1) consisted in a conversion of the carboxyl group in **2** into its benzyl ester **3** by reacting **2** with benzyl bromide in DMF, under the presence of potassium carbonate [11,17]. Subsequently, the C(3)-OH group of **3** was converted into the corresponding propargyl ether **4** using propargyl bromide, in the presence of sodium hydride in THF [18]. Note that it is important to mix **3** with sodium hydride at least 30 min before adding propargyl bromide, to enable a formation of the sodium salt of **3**. The originally used methods of etherification, employing either propargyl chloride [18] or propargylic alcohol in chloroform under the presence of Montmorillonit K [19], were not successful in this case. The propargyl ether **4** was subjected to a click reaction (Huisgen copper(I)-catalyzed 1,3-dipolar cycloaddition, CuAAC) with azidovaleric acid under a catalysis with copper(II) sulfate in a water-dichloromethane mixture, yielding **5**. TBTA was used as stabilizer of the Cu(I) oxidation state in aqueous solutions as well as acted as a rate-accelerating ligand [20,21]. To stabilize Cu(I) oxidation state is important because Cu(II) ions are harmful to this reaction due to the fact that Cu(II) ions catalyze the oxidative coupling of the alkyne substrates to give diynes as undesired by-products [20]. Sodium ascorbate acts as a reducing agent.

The main quantity of **5** was used for modification of the diosgenin molecule (**1**) by Steglich esterification with **5** under the presence of *N*,*N’*-dicyclohexylcarbodiimide (DCC) as coupling agent, and 4-(dimethylamino)pyridine (DMAP) as reaction enhancer, to get **7**. This reaction is an example of esterification that was not mediated successfully with 1-propanephosphonic anhydride (T3P), another coupling agent. T3P had often been used for formation of ester or amide bonds in our team [11]. Generally, we prefer using T3P over DCC since phosphonic acid products resulting from the T3P hydrolysis are soluble in water and are not extracted with organic solvents in contrast to dicyclohexyl urea resulting from DCC. We also tried to convert **5** to its acyl chloride with oxalyl chloride; however, this method did not yield the required product. Finally, the resulting product **7** was subjected to a high-pressure (130 kPa) hydrogenation on 10% Pd/C in an ethanol-THF mixture, yielding the target diosgenin conjugate **8**.

A deprotection of **5** and **7** was carried out in a different way than customary used procedure [10] in our laboratory (entry 1, Table 1) due to its failure. This result inspired us to search for other methods of deprotection of **5** and **7**. First, we have employed hydrogenation under atmospheric pressure (entries 2, 3 and 4 [22]) to obtain **8**. However, the hydrogenation has not been catalyzed quantitatively. Using BCl_3_ in dichloromethane (entry 5 [23]) showed no reaction, and using HBr in acetic acid (entry 6 [24]) resulted in a decomposition of the starting material. Originally, the main reason for employing hydrogenation reaction under atmospheric pressure was to prevent possible saturation of the C(20)=C(29) double bond in the betulinic acid unit of **6** and **8**. When this reaction did not furnish **6** and **8**, we have investigated hydrogenation under slightly higher pressure than atmospheric pressure, using a different palladium catalyst (entry 7). Nevertheless, it also did not catalyze the reaction in the required way. Finally, the high pressure hydrogenation procedure [17] was the successful method for deprotection of **5** and **7** and it occurred without saturating the C(20)=C(29) double bond (entries 8 and 10). However, when the pressure increased the value of 130 kPa (entry 9), saturation of the double bond appeared, indicating that 130 kPa is the critical pressure to keep the C(20)=C(29) double bond in **8** unsaturated. Saturation of the C(20)=C(29) double bond was proven by the presence of a peak belonging to the saturated product in the HR-MS spectra. Saturated product was isolated from the TLC plate and dissolved in methanol prior to measurement. It was an unexpected result, because transferring hydrogenation under atmospheric pressure was used successfully for removal of protecting benzyl group from carboxylic benzyl esters several times [10,11,13]. The reason for the failing of transferring hydrogenation in the removal of the benzyl group from **5** and **7** to get **6** and **8** can be explained by a simultaneous presence of the 1,4-disubstituted 1,2,3-triazole ring in the molecule, a phenomenon that had already been observed earlier in the literature [25]. The 1,4-disubstituted 1,2,3-triazole ring probably shows its competing effect in coordination of palladium catalyst over the target site, requiring a different approach than usually used for the successful course of the debenzylation reaction. See also Scheme S1 in the Supplementary Material.

Removal of the benzyl protecting group from **5** using the above described high pressure (130 kPa) hydrogenation was also made to prepare **6**, the other target product for pharmacological screening. HR-MS ESI supported successful removal of the benzyl protecting group of **7**, while keeping the C(20)=C(29) double bond unsaturated. In HR-MS ESI of **6** and **8**, typical adducts [M − H]^−^ were observed, C_38_H_59_O_5_N_3_ (for **6**), or C_65_H_99_O_7_N_3_ (for **8**), respectively. In HR-MS of **4**, **5**, and **7**, adducts [M − H]^+^ were identified (Table 2).

The compounds **1**–**8** were subjected to the cytotoxicity screening tests on cells of human T-lymphoblastic leukemia (CEM), cells of human breast adenocarcinoma (MCF7), cells of human cervical cancer (HeLa), and human colon carcinoma (HCT 116 cancer cell lines), using normal human fibroblasts (BJ) as reference cell lines. Doxorubicin was used as a positive control. While the target compounds **6** and **8** were inactive, due to a possible high polarity, causing a decrease in ability of the studied compounds to penetrate into the cell, **5** showed moderate to weak multifarious cytotoxicity to all cancer cell lines. Benzyl ester of the main target compound (**7**) displayed a selective cytotoxicity on CEM (6.5 ± 1.1 µM), but also weak cytotoxicity (46.2 ± 2.8 µM) towards the reference cell line BJ (Table 3).

To support the data obtained experimentally, physico-chemical and ADME parameters of the synthesized compounds have been calculated using ACD/iLabs software and databases [27] for **1**–**8** (Table 4). The data were compared with the Lipinski rule of five [28] and with the Ghose [29] rule. The Lipinski [28] and Ghose [29] rules were postulated for testing in vivo. Nevertheless, they are applicable in the screening tests in cancer cell lines, as we have proven several times in our previous papers [13,14,15]. However, deviations of experimental and calculated values have already been observed [16]. The rules describe molecular properties important for a small molecule drug pharmacokinetics in the human body, including their absorption, distribution, metabolism, and excretion (known as ADME parameters). However, the rules do not predict displaying of the pharmacological activity. The Lipinski rule of five [28] and Ghose rule [29] consider partition coefficient (log *P*, range −0.4 to +5.6), molar refractivity (range 40 to 130), molecular weight (range 180 to 500), number of atoms in the molecule (20 to 70), and polar surface area (up to 14 nm). However, the parameters calculated for some of the target compounds **1**–**8** correspond only in part to the ranges given for them by the above Lipinski [28] and Ghose [29] rules (Table 4). Despite those facts, **5** and **7**, the only cytotoxic compounds of this series, displayed cytotoxicity. For more details on calculation, explanation, and importance of physico-chemical and ADME characteristics in searching for novel potential drugs cf. Supplementary Materials.

Calculated activity on central nervous system (CNS) for **1** and **2** resulted in a finding that **1** is CNS active, while **2** appears in the area with low or no CNS activity, also made by the ACD/iLabs software [27]. The data obtained show the key parameters for assigning CNS activity, log PS*f_u, brain_ (the brain/plasma equilibration rate), log *BB* (a hybrid parameter determined by permeability, plasma and brain tissue binding and active transport mechanism; range +1.2 to −3.0), and log *PB* (the extent of brain penetration parameter, indicating if the drug might be CNS active or CNS inactive; range +2 (CNS active) to −2 (CNS inactive); cf. Table 4 and Figure S1 in the Supplementary Material). Based on the values of log PS*f_u,brain_, log *PB* and log *BB* (Table 4), **1** when liberated from **7** or **8** by their metabolic decomposition, may display certain activity on central nervous system, which may result in additional positive adaptogenic effect on organism if applied in vivo.

Aside from **1**, no one of the studied compounds (**2**–**8**) appeared to have any potential for CNS activity at all, as shown in Figure S2 in the Supplementary Material.

## 3. Material and Methods

### 3.1. General

The NMR measurements were performed either on a Bruker AVANCE 500 MHz or on a Bruker AVANCE II 600 MHz spectrometer equipped with a 5 mm TCI cryoprobe in a 5 mm tube in different solvents. The ^1^H NMR and the ^13^C NMR spectra were recorded at 499.98 MHz and 125.73 MHz (AVANCE 500 MHz) or at 600.13 MHz and 150.90 MHz (AVANCE II 600 MHz) in CDCl_3_ or CD_3_OD using tetramethylsilane (δ = 0.0 − CDCl_3_) or signal of solvent (δ = 3.31 or 49.50 for ^1^H/^13^C − CD_3_OD) as internal references. ^1^H NMR data are presented in the following order: chemical shift (δ) expressed in ppm, multiplicity (s, singlet; d, doublet; t, triplet; q, quartet; m, multiplet), number of protons, coupling constants in Hertz. For unambiguous assignment of both ^1^H and ^13^C signals, 2D NMR ^1^H,^13^C gHSQC and gHMBC spectra were measured using standard parameters sets and pulse programs delivered by producer of the spectrometer. Infrared spectra were measured with a Nicolet 205 FT-IR spectrometer (Thermofisher Scientific, Waltham, MA, USA). HR-MS was acquired on Exactive Plus Orbitrap Mass Spectrometer with HESI (coin voltage 4.5 kV, resolution 100,000 @ 1 Hz, capillary temperature 300 °C, probe temperature 30−40 °C) and were processed in Xcalibur™ 2.1 SW (ThermoFisher Scientific, Waltham, MA, USA). Samples were dissolved in methanol. See Supplementary Material for more details on HR-MS (ESI) analyses (Table 1). TLC was carried out on silica gel plates (Merck 60F_254_, Merck, Prague, Czech Republic) and the visualization was performed by both the UV detection and spraying with the methanolic solution of phosphomolybdic acid (5%) followed by heating. For column chromatography, silica gel 60 (0.063–0.200 mm) from Merck was used. All chemicals and solvents were purchased from regular commercial sources in analytical grade and the solvents were purified by general methods before use. Betulinic acid was purchased from Dr. Jan Šarek—Betulinines (www.betulinines.com). Diosgenin was purchased from Wako Chemicals (Osaka, Japan). Analytical HPLC was carried out on a TSP (Thermoseparation Products, Boston, MA, USA) instrument equipped with a ConstaMetric 4100 Bio pump and a SpectroMonitor 5000 UV DAD. The analyses of the products were performed on a reverse phase Nucleosil 120-5 C18 column (250 × 4 mm; Watrex, Prague, Czech Republic) using a methanol/water mixture (9:1, *v*/*v*) as mobile phase at 0.5 to 1.0 mL.min^−1^. The eluate was monitored at 220, 254, and 275 nm, and the UV spectra were run from 200 to 300 nm.

### 3.2. Benzyl (3β)-3-hydroxylup-20(29)-en-28-oate (***3***)

Benzyl bromide (0.65 mL; 5.48 mmol) was added to a solution of betulinic acid (**2**; 1.0 g; 2.19 mmol) in acetone (40 mL), in a presence of potassium carbonate (0.45 g; 3.28 mmol), and the reaction mixture was stirred at r.t. for 24 h. The mixture was worked-up by diluting it with chloroform and extracted with water. Organic layer was dried with sodium sulfate, then the solvent was evaporated under reduced pressure, and the residue was purified by chromatography using chloroform/EtOH mixture as mobile phase yielding **3** as white powder in a 99% yield (1.19 g). ^1^H-NMR (499.98 MHz, CDCl_3_): δ [ppm] 0.61 (dd, 1H, *J* = 2.5; 11.3 Hz, H-5), 0.70 (s, 3H, H-23), 0.71 (s, 3H, H-24), 0.75 (d, 3H, *J* = 0.7 Hz, H-25), 0.90 (d, 3H, *J* = 0.6 Hz, H-26), 0.91 (s, 3H, H-27), 1.05 (dt, 2H, *J* = 3.0; 3.0; 13.2 Hz, H-21), 1.63 (dd, 3H, *J* = 0.7; 1.4 Hz, H-30), 2.14 (ddd, 1H, *J* = 3.7; 11.6; 12.9 Hz, H-13), 2.23 (dt, 2H, *J* = 3.4; 3.4; 12.5 Hz, H-16), 2.97 (dt, 1H, *J* = 4.6; 11.0; 11.0 Hz, H-19), 3.13 (dd, 1H, *J* = 4.9; 11.4 Hz, H-3), 4.55 (dq, 2H, *J* = 1.4; 1.4; 1.4; 2.4 Hz, H-29), 4.68 (dq, 2H, *J* = 0.7; 0.7; 0.7; 2.4 Hz, H-29), 5.05 (d, 2H, *J* = 12.3 Hz, H-1′), 5.10 (d, 2H, *J* = 12.3 Hz, H-1′), 7.24–7.35 (m, 2H, H-3′,H-4′,H-5′). ^13^C-NMR (125.73 MHz, CDCl_3_): δ [ppm] 14.66 (q, C-27), 15.34 (q, C-24), 15.81 (q, C-25), 16.11 (q, C-26), 18.27 (t, C-6), 19.35 (q, C-29), 20.85 (t, C-11), 25.51 (t, C-12), 27.39 (t, C-2), 27.96 (q, C-23), 29.55 (t, C-21), 30.56 (t, C-15), 32.10 (t, C-16), 34.28 (t, C-22), 36.92 (t, C-7), 37.15 (s, C-10), 38.18 (d, C-13), 38.69 (t, C-1), 38.83 (s, C-4), 40.62 (s, C-8), 42.37 (s, C-14), 46.91 (d, C-19), 49.42 (d, C-18), 50.53 (d, C-9), 55.32 (d, C-5), 56.53 (s, C-17), 65.71 (t, C-1′), 78.96 (d, C-3), 109.56 (t, C-30), 128.04 (d, C-5′), 128.22 (d, C-4′), 128.47 (d, C-3′), 136.47 (s, C-2′), 150.57 (s, C-20), 175.80 (s, C-28). IR (KBr): [cm^−1^] 3533 s (O-H) stretching, 2940 s (ring system, sp^3^, C-H) stretching, 1692 s (C=O) stretching, 1647 w (benzene, C=C) stretching, 1453 m (benzene, C=C) stretching, 1184, 757, 699. HR-MS (ESI) *m*/*z* 547.4172, calcd. 547.4146 for C_37_H_55_O_3_.

### 3.3. Benzyl (3β)-3-(prop-2-yn-1-yloxy)lup-20(29)-en-28-oate (***4***)

A portion of sodium hydride (0.48 g; 20.44 mmol) was added to a solution of **3** (0.325 g; 0.60 mmol) in THF (15 mL), and the mixture was stirred at r.t. for 30 min. Then, an additional portion of sodium hydride (0.48 g; 20.44 mmol) was added, and the mixture was stirred for an additional 30 min. Propargyl bromide (0.176 mL; 2.32 mmol) was added, and the mixture was stirred at r.t. for 24 h. Then, additional propargyl bromide (0.137 mL; 1.80 mmol) was added, and the mixture was stirred at r.t. for an additional 24 h. The reaction was then worked-up by extraction with chloroform (4 × 15 mL). The extract was washed with water, dried over sodium sulfate, filtered, and the solvent was evaporated under reduced pressure. The residue was purified by column chromatography on silica gel, using petroleum ether/chloroform as mobile phase, yielding **4** in a 96% (0.336 g) yield. ^1^H-NMR (499.98 MHz, CDCl_3_): δ [ppm] 0.61 (dd, 1H, *J* = 2.2; 11.3 Hz, H-5), 0.68 (s, 3H, H-23), 0.69 (s, 3H, H-24), 0.73 (s, 3H, H-25), 0.87 (d, 3H, *J* = 0.6 Hz, H-26), 0.89 (s, 3H, H-27), 1.02 (dt, 2H, *J* = 3.2; 3.2; 13.1 Hz, H-21), 1.61 (dd, 3H, *J* = 0.7; 1.4 Hz, H-30), 2.11 (ddd, 1H, *J* = 3.7; 11.5; 12.9 Hz, H-13), 2.21 (dt, 2H, *J* = 2.9; 2.9; 12.3 Hz, H-16), 2.29 (t, 1H, *J* = 2.4 Hz, H-3′), 2.92 (dd, 1H, *J* = 4.4; 11.8 Hz, H-3), 2.95 (dt, 1H, *J* = 5.8; 11.2; 11.2 Hz, H-19), 4.07 (dd, 2H, *J* = 2.4; 16.0 Hz, H-1′), 4.15 (dd, 2H, *J* = 2.4; 16.0 Hz, H-1′), 4.52 (dq, 2H, *J* = 1.4; 1.4; 1.4; 2.4 Hz, H-29), 4.66 (dq, 2H, *J* = 0.7; 0.7; 0.7; 2.4 Hz, H-29), 5.02 (d, 2H, *J* = 12.3 Hz, H-4′), 5.07 (d, 2H, *J* = 12.3 Hz, 4′), 7.23–7.32 (m, 2H, H-6′, H-7′, H-8′). ^13^C-NMR (125.73 MHz, CDCl_3_): δ [ppm] 14.62 (q, C-27), 15.82 (q, C-24), 16.10 (q, C-25), 16.17 (q, C-26), 18.21 (t, C-6), 19.37 (q, C-29), 20.89 (t, C-11), 25.54 (t, C-12), 27.96 (t, C-2), 29.54 (q, C-23), 30.17 (t, C-21), 30.57 (t, C-15), 32.10 (t, C-16), 34.29 (t, C-22), 36.93 (t, C-7), 37.11 (t, C-10), 38.18 (d, C-13), 38.53 (t, C-1), 38.56 (s, C-4), 40.67 (s, C-8), 42.37 (s, C-14), 46.92 (d, C-19), 49.44 (d, C-18), 50.52 (d, C-9), 55.89 (d, C-5), 56.38 (t, C-1′), 56.53 (s, C-17), 65.71 (t, C-4′), 73.39 (d, C-3), 80.83 (d, C-3′), 80.95 (s, C-2′), 109.55 (t, C-30), 128.04 (d, C-8′), 128.23 (d, C-7′), 128.47 (d, C-6′), 136.47 (s, C-5′), 150.60 (s, C-20), 175.79 (s, C-28). IR (KBr): [cm^−1^] 3305 m (sp, C-H) stretching, 2957 s (ring system, sp^3^, C-H) stretching, 1715 s (C=O), 1644 w (benzene, C=C) stretching, 1453 m (benzene, C=C), 1128, 1080, 755, 628. HR-MS (ESI) *m*/*z* 585.4289, calcd. 585.4302 for C_40_H_57_O_3_.

### 3.4. 5-[4-({[(3β)-28-(Benzyloxy)-28-oxolup-20(29)-en-3-yl]oxy}-methyl)-1H-1,2,3-triazol-1-yl]pentanoic acid (***5***)

5-Azidovaleric acid (61.27 mg; 0.43 mmol), sodium ascorbate (16.94 mg; 0.09 mmol) and a solution of CuSO_4_·5H_2_O with TBTA in water (7.61 mL; 0.89 mmol) was added to a solution of 4 (200 mg; 0.34 mmol) in CH_2_Cl_2_ (5 mL), and the mixture was stirred at r.t. for 30 h. The mixture was worked-up by extraction with methylene chloride, the extract was washed with water, and dried over sodium sulfate. After filtration, the solvent was evaporated under reduced pressure, and the residue was purified by column chromatography on silica gel, using petroleum ether/chloroform mixture as mobile phase, yielding **5** in a 95% yield (0.26 g). ^1^H-NMR (499.98 MHz, CDCl_3_): δ [ppm] 0.58 (dd, 1H, *J* = 2.3; 11.2 Hz, H-5), 0.68 (s, 3H, H-23), 0.68 (s, 3H, H-24), 0.73 (s, 3H, H-25), 0.81 (s, 3H, H-26), 0.86 (s, 3H, H-27), 1.02 (dt, 2H, *J* = 3.2; 3.2; 13.5 Hz, H-21), 1.52 (t, 1H, *J* = 11.4 Hz, H-18), 1.53–1.65 (m, 2H, H-5′, H-6′), 1.61 (dd, 3H, *J* = 0.7; 1.4 Hz, H-30), 2.10 (ddd, 1H, *J* = 3.6; 11.5; 12.8 Hz, H-13), 2.20 (dt, 2H, *J* = 3.1; 3.1; 12.5 Hz, H-16), 2.33 (t, 2H, *J* = 7.3 Hz, H-7′), 2.87 (dd, 1H, *J* = 4.4; 11.8 Hz, H-3), 2.95 (dt, 1H, *J* = 4.8; 11.0; 11.0 Hz, H-19), 4.30 (t, 2H, *J* = 7.1 Hz, H-4′), 4.49 (dd, 2H, *J* = 0.6; 12.5 Hz, H-1′), 4.53 (dq, 2H, *J* = 1.4; 1.4; 1.4; 2.4 Hz, H-29), 4.65 (dq, 2H, *J* = 0.7; 0.7; 0.7; 2.4 Hz, H-29), 4.70 (dd, 2H, *J* = 0.6; 1.2 Hz, H-1′), 5.02 (d, 2H, *J* = 12.3 Hz, H-9′), 5.07 (d, 2H, *J* = 12.3 Hz, H-9′), 7.22–7.32 (m, 2H, H-11′, H-13′), 7.44 (s, 1H, H-3′). ^13^C-NMR (125.73 MHz, CDCl_3_): δ [ppm] 14.61 (q, C-27), 15.81 (q, C-24), 16.12 (q, C-25), 16.25 (q, C-26), 18.20 (t, C-6), 19.34 (q, C-29), 20.87 (t, C-11), 21.52 (t, C-6′), 22.89 (t, C-5′), 25.51 (t, C-12), 27.99 (q, C-23), 28.19 (t, C-2), 29.51 (t, C-15), 30.55 (t, C-21), 32.09 (t, C-16), 32.97 (t, C-7′), 34.26 (t, C-22), 36.92 (t, C-7), 37.11 (s, C-10), 38.17 (d, C-13), 38.50 (t, C-1), 38.78 (s, C-4), 40.65 (s, C-8), 42.35 (s, C-14), 46.92 (d, C-19), 49.42 (d, C-18), 49.85 (t, C-4′), 50.47 (d, C-9), 55.70 (d, C-5), 56.53 (s, C-17), 63.18 (t, C-1′), 65.70 (t, C-9′), 86.61 (d, C-3), 109.57 (t, C-30), 122.09 (d, C-3′), 128.02 (d, C-13′), 128.21 (d, C-12′), 128.46 (d, C-11′), 136.46 (s, C-10′), 146.55 (s, C-2′), 150.56 (s, C-20), 175.81 (s, C-28), 177.46 (s, C-8′). IR (KBr): [cm^−1^] 2945 s br (ring system, sp^3^ C-H and O-H overlap) stretching, 1732 s (C=O) stretching, 1642 w (benzene, C=C) stretching, 1453 m (benzene, C=C) stretching, 1130. HR-MS (ESI) *m*/*z* 728.4987, calcd. 728.4997 for C_45_H_66_O_5_N_3_.

### 3.5. (3β)-3-{[1-(4-Carboxybutyl)-1H-1,2,3-triazol-4-yl]methoxy}lup-20(29)-en-28-oic Acid (***6***)

The compound **5** (75 mg, 0.105 mmol) was subjected to a removal of the benzyl protecting group by hydrogenation on a 10% Pd/C (75 mg, 0.705 mmol) in a THF/ethanol (1:1) mixture (8 mL) at 130 kPa at r.t. for 30 min. After filtering the mixture off to remove the catalyst, and evaporation of the solvent, the residue was purified by column chromatography, using petroleum ether/ethyl acetate as mobile phase, yielding **6** in a 92% yield (61.3 mg). ^1^H-NMR (600.13 MHz, CDCl_3_): δ [ppm] 0.61 (dd, 1H, *J* = 1.9; 11.4 Hz, H-5), 0.68 (s, 3H, H-25), 0.75 (s, 3H, H-24), 0.81 (d, 3H, *J* = 0.6 Hz, H-23), 0.84 (d, 3H, *J* = 0.8 Hz, H-26), 0.89 (d, 3H, *J* = 0.5 Hz, H-27), 1.54 (t, 1H, *J* = 11.4 Hz, H-18), 1.58 (dd, 3H, *J* = 0.7; 1.4 Hz, H-30), 2.09 (ddd, 1H, *J* = 3.8; 11.8; 12.8 Hz, H-13), 2.19 (ddd, 2H, *J* = 3.2; 3.7; 13.1 Hz, H-16), 2.34 (t, 2H, *J* = 7.2 Hz, H-7′), 2.89 (dd, 1H, *J* = 4.4; 11.7 Hz, H-3), 2.92 (dt, 1H, *J* = 4.9; 11.0; 11.5 Hz, H-19), 4.31 (dt, 2H, *J* = 1.0; 7.0; 7.0 Hz, H-4′), 4.48 (dd, 2H, *J* = 0.7; 12.5 Hz, H-1′), 4.54 (dq, 2H, *J* = 1.4; 1.4; 1.4; 2.4 Hz, H-29), 4.67 (dq, 2H, *J* = 0.7; 0.7; 0.7; 2.4 Hz, H-29), 4.72 (dd, 2H, *J* = 0.6; 12.5 Hz, H-1′), 7.46 (s, 1H, H-3′). ^13^C-NMR (150.91 MHz, CDCl_3_): δ [ppm] 14.63 (q, C-27), 16.10 (q, C-24), 16.13 (q, C-26), 16.24 (q, C-25), 18.21 (t, C-6), 19.36 (q, C-29), 20.84 (t, C-6′), 21.51 (t, C-11), 22.93 (t, C-5′), 25.45 (t, C-12), 28.01 (q, C-23), 29.52 (t, C-2), 29.68 (t, C-15), 30.56 (t, C-21), 32.13 (t, C-16), 33.14 (t, C-7′), 34.25 (t, C-22), 37.05 (t, C-7), 37.14 (s, C-10), 38.40 (d, C-13), 38.50 (t, C-1), 38.80 (s, C-4), 40.67 (s, C-8), 42.38 (s, C-14), 46.90 (t, C-19), 49.28 (d, C-18), 49.91 (t, C-4′), 50.38 (d, C-9), 55.70 (d, C-5), 56.42 (s, C-17), 63.31 (t, C-1′), 86.73 (d, C-3), 109.69 (t, C-30), 122.10 (d, C-3′), 146.60 (s, C-2′), 150.39 (s, C-20), 178.37 (s, C-28), 182.46 (s, C-8′). IR (KBr): [cm^−1^] 2944 s, (ring system, sp^3^ C-H and O-H overlap) stretching, 1705 s (C=O) stretching. HR-MS (ESI) *m*/*z* 636.4413, calcd. 636.4382 for C_38_H_59_O_5_N_3_.

### 3.6. Benzyl (3β)-3-[(1-{5-oxo-5-[(3β,25R)-spirost-5-en-3-yloxy]pentyl}-1H-1,2,3-triazol-4-yl)methoxy]lup-20(29)-en-28-oate (***7***)

DCC (167.0 mg, 0.81 mmol) and DMAP (152.5 mg, 1.25 mmol) were added to a solution of **5** (168 mg, 0.23 mmol) in methylene chloride (10 mL) under stirring. Then, diosgenin (**1**; 143 mg, 0.35 mmol) was added, and stirring continued at r.t. for 24 h. The crude product was purified by column chromatography on silica gel using petroleum ether/ethyl acetate as mobile phase, yielding white, crystalline **7** in a 72% yield (186 mg). ^1^H-NMR (600.13 MHz, CDCl_3_): δ [ppm] 0.64 (dd, 1H, *J* = 2.1; 11.2 Hz, H-5), 0.74 (s, 3H, H-23), 0.74 (s, 3H, H-19″), 0.76 (s, 3H, H-25), 0.78 (d, 3H, H-27″), 0.86 (s, 3H, H-24), 0.92 (s, 3H, H-26), 0.96 (d, 3H, *J* = 7.0 Hz, H-21″), 1.02 (s, 3H, H-27), 1.02 (s, 3H, H-18″), 1.67 (dd, 3H, *J* = 0.7; 1.4 Hz, H-30), 2.16 (ddd, 1H, *J* = 3.5; 11.6; 12.8 Hz, H-13), 2.26 (dt, 2H, *J* = 3.1; 3.1; 12.7 Hz, H-16), 2.31 (t, 2H, *J* = 7.3 Hz, H-12′), 2.93 (dd, 1H, *J* = 4.3; 11.7 Hz, H-3), 3.01 (dt, 1H, *J* = 4.9; 10.9; 10.9 Hz, H-19), 3.37 (t, 2H, *J* = 11.0 Hz, H-26″), 3.44–3.50 (m, 2H, H-26″), 3.44–3.50 (m, 1H, H-20″), 4.35 (t, 2H, *J* = 7.0 Hz, H-9′), 4.40 (ddd, 1H, *J* = 6.5; 7.6; 8.5 Hz, H-16″), 4.54 (dd, 2H, *J* = 0.5; 12.5 Hz, H-6′), 4.58 (dq, 2H, *J* = 1.4; 1.4; 1.4; 2.3 Hz, H-29), 4.59 (dddd, 2H, *J* = 4.3; 6.5; 10.6; 11.4 Hz, H-3″), 4.71 (dq, 2H, *J* = 0.7; 0.7; 0.7; 2.3 Hz, H-29), 4.76 (dd, 2H, *J* = 0.5; 12.5 Hz, H-6′), 5.08 (dd, 2H, *J* = 0.5; 12.5 Hz, H-1′), 5.13 (dd, 2H, *J* = 0.5; 12.5 Hz, H-1′), 5.36 (dt, 2H, *J* = 0.9; 0.9; 5.3 Hz, H-6″), 7.28–7.37 (m, 2H, H-3′-H-5′). ^13^C-NMR (150.91 MHz, CDCl_3_): δ [ppm] 14.51 (q, C-21″), 14.62 (q, C-27), 16.12 (q, C-25), 16.25 (q, C-18″), 16.26 (q, C-24), 17.12 (q, C-27″), 18.22 (t, C-6), 19.32 (q, C-29), 19.34 (q, C-19″), 20.77 (t, C-11″), 20.87 (t, C-11), 21.84 (t, C-10′), 22.89 (t, C-11′), 23.82 (t, C-2), 25.51 (t, C-12), 25.82 (q, C-26), 27.74 (q, C-23), 28.78 (t, C-24″), 29.53 (t, C-21), 29.62 (t, C-23″), 30.28 (d, C-8″), 30.56 (t, C-15), 31.37 (t, C-2″), 31.37 (d, C-25″), 31.81 (t, C-7″), 32.02 (t, C-15″), 32.10 (t, C-16), 33.89 (t, C-12′), 33.89 (t, C-12″), 34.28 (t, C-22), 36.70 (s, C-10″), 36.91 (s, C-10), 36.93 (t, C-7), 37.12 (t, C-1″), 38.08 (s, C-4), 38.18 (d, C-13), 38.51 (t, C-1), 40.66 (s, C-8), 40.83 (s, C-13″), 41.59 (t, C-4″), 42.36 (s, C-14), 46.93 (d, C-19), 49.21 (d, C-20″), 49.43 (d, C-18), 49.90 (t, C-9′), 49.91 (d, C-9″), 50.48 (d, C-9), 55.71 (d, C-5), 56.41 (d, C-14″), 56.53 (s, C-17), 62.05 (d, C-17″), 63.22 (t, C-6′), 65.70 (t, C-1′), 66.83 (t, C-26″), 74.01 (d, C-3″), 80.78 (d, C-16″), 86.49 (d, C-3), 109.27 (s, C-22″), 109.58 (t, C-30), 122.01 (d, C-8′), 122.44 (d, C-6″), 128.03 (d, C-5′), 128.22 (d, C-4′), 128.46 (d, C-3′), 136.47 (s, C-2′), 139.56 (s, C-5″), 146.55 (s, C-7′), 150.57 (s, C-20), 175.80 (s, C-28). IR (KBr): [cm^−1^] 2945 s, (ring system, sp^3^, C-H) stretching, 1731 s (C=O), 1626 m (alkene, C=C) stretching, 1456 m (benzene, C=C) stretching, 1128. HR-MS (ESI) *m*/*z* 1124.8025, calcd. 1124.8023 for C_72_H_105_O_7_N_3_.

### 3.7. (3β)-3-[(1-{5-Oxo-5-[(3β,25R)-spirost-5-en-3-yloxy]pentyl}-1H-1,2,3-triazol-4-yl]methoxy}lup-20(29)-en-28-oic Acid (***8***)

The benzyl ester **7** (186.3 mg, 0.166 mmol) was subjected to a hydrogenation on a 10% Pd/C (118.1 mg, 0.111 mmol) in a THF/ethanol (1:1; 10 mL) mixture at 130 kPa at r.t. for 40 min. The mixture was then filtered off and the product was purified by column chromatography on silica gel, using a chloroform/ethanol mixture (200:1) as mobile phase, yielding the product **8** in an 87% yield (149 mg). ^1^H-NMR (600.13 MHz, CDCl_3_): δ [ppm] 0.61 (dd, 1H, *J* = 1.9; 11.4 Hz, H-5), 0.69 (s, 3H, H-19′’), 0.72 (d, 3H, *J* = 6.4 Hz, H-27′’), 0.75 (s, 3H, H-18′’), 0.76 (s, 3H, H-25), 0.81 (s, 3H, H-23), 0.86 (s, 3H, H-24), 0.89 (s, 3H, H-26), 0.89 (s, 3H, H-27), 0.90 (d, 3H, *J* = 7.0 Hz, H-21′’), 0.96 (s, 3H, H-30), 2.12 (ddd, 1H, *J* = 3.8; 11.8; 12.8 Hz, H-13), 2.28 (ddd, 2H, *J* = 2.8; 4.6; 12.8 Hz, H-16), 2.88 (dd, 1H, *J* = 4.4; 11.7 Hz, H-3), 2.94 (dt, 1H, *J* = 5.0; 10.9; 10.9, H-19), 3.31 (t, 1H, *J* = 11.0 Hz, H-26′’), 3.41 (ddt, 1H, *J* = 1.8; 1.8; 5.0; 11.0 Hz, H-26′’), 4.34 (ddd, 1H, *J* = 6.4; 7.3; 8.5 Hz, H-16′’), 4.50 (d, 1H, *J* = 12.6 Hz, H-1′), 4.53 (dddd, 1H, *J* = 4.6; 6.3; 10.5; 11.7 Hz, H-3′’), 4.54 (dq, 1H, *J* = 3 × 1.4; 2.3 Hz, H-29), 4.67 (bd, 1H, *J* = 2.3 Hz, H-29), 4.70 (d, 1H, *J* = 12.6 Hz, H-1′), 5.30 (dt, 1H, *J* = 1.9; 1.9; 5.3 Hz, H-6′’), 7.45 (s, 1H, H-3′). ^13^C-NMR (150.91 MHz, CDCl_3_): δ [ppm] 14.48 (q, C-21′’), 14.65 (q, C-27), 16.08 (q, C-26), 16.17 (q, C-25), 16.27 (q, C-24), 16.27 (q, C-18′’), 17.12 (q, C-27′’), 18.23 (t, C-6), 19.33 (q, C-29), 19.36 (q, C-19′’), 20.81 (t, C-11′’), 20.87 (t, C-11), 21.83 (t, C-5′), 22.93 (t, C-2), 22.93 (t, C-6′), 25.47 (t, C-12), 28.03 (q, C-23), 28.77 (t, C-24′’), 29.35 (t, C-21), 29.56 (t, C-23′’), 30.27 (d, C-8′’), 30.56 (t, C-15), 31.36 (t, C-2′’), 31.36 (d, C-25′’), 31.80 (t, C-7′’), 32.01 (t, C-15′’), 32.17 (t, C-16), 33.68 (t, C-7′), 33.68 (t, C-12′’), 34.29 (t, C-22), 36.70 (s, C-10′’), 36.93 (s, C-10), 37.13 (t, C-7), 37.15 (t, C-1′’), 38.10 (s, C-4), 38.50 (d, C-13), 38.80 (t, C-1), 40.24 (s, C-8), 40.70 (s, C-13′’), 41.61 (t, C-4′’), 42.41 (s, C-14), 46.91 (d, C-19), 49.26 (d, C-20′’), 49.86 (d, C-18), 49.93 (t, C-4′), 49.93 (d, C-9′’), 50.42 (d, C-5), 55.70 (d, C-5), 56.25 (d, C-14′’), 56.42 (s, C-17), 62.00 (d, C-17′’), 63.26 (t, C-1′), 66.83 (t, C-26′’), 74.02 (d, C-3′’), 80.82 (d, C-16′’), 86.54 (d, C-3), 109.35 (s, C-22′’), 109.68 (t, C-30), 122.01 (d, C-3′), 122.45 (d, C-6′’), 139.56 (s, C-5′’), 146.64 (s, C-2′), 150.43 (s, C-20), 172.35 (s, C-8′), 179.92 (s, C-28). IR (KBr): [cm^−1^] 3322 m (O-H) stretching, 2928 s (ring system, sp^3^, C-H) streching, 1731 s (C=O) stretching, 1620 m (C=C) stretching → alkene, 1243, 1088. HR-MS (ESI) *m*/*z* 1033.7533, calcd. 1033.7483 for C_65_H_99_O_7_N_3_.

### 3.8. Cell Cultures

The screening cell lines, T-lymphoblastic leukemia (CEM), cervical carcinoma (HeLa), breast carcinoma (MCF7), and human foreskin fibroblasts (BJ) were obtained from the American Type Culture Collection (Manassas, VA, USA). Cells were cultured in DMEM (Dulbecco’s Modified Eagle Medium, Sigma Aldrich, Darmstadt, Germany). Media used were supplemented with 10% fetal bovine serum, 2 mM l-glutamine, and 1% penicillin-streptomycin (all from Sigma Aldrich, Darmstadt, Germany). The cell lines were maintained under standard cell culture conditions at 37 °C and 5% CO_2_ in humid environment. Cells were subcultured twice or three times a week using the standard trypsinization procedure.

### 3.9. Cytotoxicity Screening Tests

Description of the experimental procedure used in cytotoxicity assay was already published [11]. The IC_50_ values in CEM, MCF7, HeLa, HCT 116, and BJ measured with the compounds **1**–**8** after 72 h of treatment are shown in Table 3.

## 4. Conclusions

We have developed an easy and efficient method for constructing the ester bond to connect highly hindered and/or low reactive components. Several coupling reagents were studied and their application for a given synthesis has been optimized. Different methods of removal of benzyl protecting group were tested under different hydrogenation conditions and the main focus was given on prevention of any double bond reduction. Removal of benzyl protecting group in **5** and **7** in the presence of a 1,4-disubstituted 1,2,3-triazole ring in the molecule has seemed to be a competing element for coordination of palladium, disabling the required palladium-catalyzed reaction. Palladium-catalyzed removal of the benzyl group is enabled only under high pressure (130 kPa), and this pressure value sets as a critical one. In the cytotoxicity screening tests, we found that less polar conjugates display higher cytotoxicity than more polar ones (cf. compound **5** *versus* **6**, and compound **7** *versus* **8**).

Diosgenin (**1**), betulinic acid (**2**) and the synthetic intermediates **3** and **4** were not cytotoxic, i.e., their IC_50_ values are higher than 50 µM against all tested cell lines. This finding resulted in a conclusion that the synthesized type of a conjugate is capable of enhancing the pharmacological activity of its components, and they act in a synergy. Moreover, selectivity towards cancer cells was observed, which augments the importance of the target conjugates. In turn, conjugation of diosgenin (**1**) with betulinic acid (**2**) in **7** or **8** resulted in the theoretical in silico calculation showing no CNS activity of the conjugates **7** and **8** in comparison with that of **1** (cf. Supplementary Material).

Even if most of the physico-chemical and ADME parameters calculated for **1**–**8** do not correspond to some of their limits given by the Lipinski [28] and Ghose [29] rules, **5** still showed selective cytotoxicity and **7** showed medium to weak multifarious cytotoxicity. This finding clearly demonstrates that in silico calculations should always be accompanied by the experimental work for comparing both approaches and for confronting theoretical calculations with practical results. They are applicable in the screening tests in cancer cell lines, as we have proven several times in our previous papers [13,14,15]. In turn, we have already observed deviation of the experimental and calculated values [16]. Diosgenin (**1**) displays certain potential for a weak CNS active drug (cf. Supplementary Material), which finding is in a good agreement with identifying **1** as an adaptogen [3].

The importance of phytochemicals, namely terpenoids, saponins, polyphenols, and alkaloids, has recently been reviewed and evaluated [30]. Plant products represent an important source of compounds capable of displaying a broad variety of effects on human health, among which cancer chemotherapy is the most important. This review paper [30] also supports findings and conclusions presented herewith.

## Figures and Tables

**Table 1:** Applied methods of conversion of **7** to **8**.

Entry	Catalyst	Equiv.	Solvent	H-donor	Press.	Temp.	Result ^a^
1	10% Pd/C	0.67 [10]	EtOH/THF	1,4-cyclohexadiene	atm	25 °C	no rxn ^b^
2	10% Pd/C	0.67	EtOH/THF	H_2_	atm	25 °C	n. c. ^c^
3	PdO	2.0	EtOH/EtOAc	H_2_	atm	25 °C	n. c. ^c^
4	Pd(OAc)_2_	0.05 [22]	CH_2_Cl_2_	H_2_	atm	25 °C	n. c. ^c^
5	BCl_3_	1.05 [23]	CH_2_Cl_2_	-	atm	25 °C	no rxn ^b^
6	HBr	1.0 [24]	AcOH	-	atm	60 °C	dec. ^d^
7	Pd/BaSO_4_	0.05	EtOH/dioxane	H_2_	101.3 kPa	25 °C	n. c. ^c^
8	10% Pd/C	0.67	EtOH/THF	H_2_	<130 kPa	25 °C	success
9	10% Pd/C	0.67	EtOH/THF	H_2_	>130 kPa	25 °C	sat. ^e^
10	10% Pd/C	0.67	EtOH/THF	H_2_	130 kPa	25 °C	success

^a^ result of the course of the reaction, ^b^ no reaction observed, ^c^ reaction not completed, ^d^ decomposition (cleavage of the molecule), ^e^ saturation of the C(20)=C(29) double bond.

**Table 2:** HR-MS ESI of compounds **3**–**8.**

Compound	Measured Mass	Calculated Formula	Theoretical Mass	Mass Error
	(*m*/*z*)		(*m/z*)	(ppm)
**3**	547.4172	C_37_H_55_O_3_	547.4146	+4.87
**4**	585.4289	C_40_H_57_O_3_	585.4302	+2.19
**5**	728.4987	C_45_H_66_O_5_N_3_	728.4997	−1.31
**6**	636.4413	C_38_H_59_O_5_N_3_	636.4382	−4.85
**7**	1124.8025	C_72_H_105_O_7_N_3_	1124.8025	+0.02
**8**	1033.7533	C_65_H_99_O_7_N_3_	1033.7483	+4.88

**Table 3:** Cytotoxicity screening tests (IC_50_ values (μM), 72 h).

Compound	MW	Cytotoxicity (IC_50_ (μM))				
		CEM ^a^	MCF7 ^b^	HeLa ^c^	HCT 116 ^d^	BJ ^e^
**1**	414.62	>50	>50	>50	>50	>50
**2**	456.70	>50	>50	>50	>50	>50
**3**	546.82	>50	>50	>50	>50	>50
**4**	584.87	>50	>50	>50	>50	>50
**5**	728.02	15.8 ± 0.6	49.5 ± 0.7	20.3 ± 4.4	19.4 ± 2.1	>50
**6**	637.89	>50	>50	> 50	>50	>50
**7**	1124.62	6.5 ± 1.1	>50	> 50	>50	46.2 ± 2.8
**8**	1034.50	>50	>50	> 50	>50	>50
Doxorubicin	543.53	NT ^f^	0.273 ± 0.019	0.868 ± 0.054	0.524 ± 0.177	0.278 ± 0.036

^a^ CEM, cells of human T-lymphoblastic leukemia; ^b^ MCF7, cells of human breast adenocarcinoma; ^c^ HeLa, cells of human cervical cancer; ^d^ HCT 116, human colon carcinoma; ^e^ BJ, normal human fibroblasts; ^f^ NT = not tested in our team; however, IC_50_ value (CEM) 0.02 + 0.00 µM (ref. [26]).

**Table 4:** Physico-chemical and absorption, distribution, metabolism, and excretion (ADME) parameters of **1**–**8** calculated using the ACD/iLabs software [27].

Compd. or Recom. Range	MW	Physico-Chemical Parameters ^a,b^				ADME Parameters ^c^					
		log *P*	log *D* (pH 7.4)	log *S* (pH 7.4)	H_acc_/H_don_/n.m.b.	Bioav. (%)	log *PS* * *f_u,brain_*	log *PS*	log *PB*	log *BB*	PPB (%)
**1**	414.62	+6.01	+6.01	−5.65	3/1/0	30–70	−3.5	−1.2	+0.29	+0.28	98.97
**2**	456.70	+6.26	+3.77	−3.34	3/2/2	30–70	−4.4	−2.6	−0.35	−0.20	99.29
**3**	546.82	+8.92	+8.92	−8.10	3/1/5	<30	−5.2	−2.9	−0.58	−0.58	99.87
**4**	584.87	+9.87	+9.87	−9.22	3/0/8	<30	−5.7	−3.4	−0.92	−0.92	99.94
**5**	728.01	+9.12	+6.62	−6.07	8/1/13	<30	−6.9	−5.1	−2.00	−2.00	99.93
**6**	637.89	+6.65	+1.96	−2.21	8/2/10	30–70	−5.2	−3.4	−0.88	−0.88	99.79
**7**	1124.62	+15.11	+15.11	−10.72	10/0/15	<30	−10.5	−8.1	−2.00	−2.00	100.00
**8**	1034.50	+13.15	+10.66	−7.51	10/1/12	<30	−10.1	−8.3	−2.00	−2.00	100.00
recom. range	180/500	−0.4/+5.6	-	−6.5/+0.5	10/5/-	-	-	-	−1.5/+1.5	−3.0/+1.2	-

^a^ log *P*—partition coefficient; log *D*—distribution coefficient; log *S*—predicted aqueous solubility; H_acc_/H_don_/n.m.b. = number of H-bond acceptors/number of H-bond donors/number of movable bonds; ^b^ Lipinski rule [28]: MW = max. 500, log *P* = max. 5.0, H_acc_/H_don_/n.m.b. = max. 10/max. 5/−, and Ghose rule [29]: MW = max. 500, log *P* = max. 5.6; ^c^ bioav. = bioavailability—the degree of availability of a chemical by the target tissue; log *PS* * *f_u,brain_*—the brain/plasma equilibration rate, the parameter that is a mathematical modeling parameter based on time required for reaching brain equilibrium; log *PS*—logarithm of the permeability-surface area coefficient; log *PB*—the extent of brain penetration parameter; log *BB*—a hybrid parameter determined by permeability, plasma and brain tissue binding, and active transport mechanism; PPB—plasma protein binding.

## Supplementary Materials

Electronic Supplementary Material is available. Details on a graphical image of conversion of **7** to **8**, and physico-chemical and ADME parameters of the target compounds are presented therein, and it can be found online. Scheme S1: Applied methods of conversion of **7** to **8**, graphical image. Figure S1: A comparison of the calculated position of diosgenin (**1**; green dot) and betulinic acid (**2**; green dot) in the diagram of the currently used CNS active drugs (blue dots) and CNS inactive drugs (orange dots). Figure S2: Calculated CNS activity of **5** (A), **6** (B), **7** (C) and **8** (D). Green dots are located in the left bottom corners of the pictures (for A, C and D).
