# Supplementary material for: Synthesis and Pharmacological Effects of Diosgenin–Betulinic Acid Conjugates
Source: Molecules. 2020 Aug 3;25(15):3546. doi: 10.3390/molecules25153546 (PMC7435711; doi:10.3390/molecules25153546)
Supplement: Supplementary file 1 [file molecules-25-03546-s001.pdf]

Supplementary Material

Synthesis and Pharmacological Effects of Diosgenin–Betulinic Acid Conjugates

Zülal Özdemir,[1,2] Michaela Rybková,[2] Martin Vlk,[1,3] David Šaman,[4] Lucie Rárová,[5] and Zdeněk Wimmer [1,2,]*

[1] Institute of Experimental Botany of the Czech Academy of Sciences, Isotope Laboratory, Vídeňská 1083, 14220 Prague 4, Czech Republic;

[2] University of Chemistry and Technology in Prague, Department of Chemistry of Natural Compounds, Technická 5, 16628 Prague 6, Czech Republic;

[3] Czech Technical University in Prague, Faculty of Nuclear Sciences and Physical Engineering, Břehová 7, 11519 Prague 1, Czech Republic;

[4] Institute of Organic Chemistry and Biochemistry of the Czech Academy of Sciences, Flemingovo náměstí 2, 16610 Prague 6, Czech Republic.

[5] Laboratory of Growth Regulators, Faculty of Science, Palacký University, and Institute of Experimental Botany of the Czech Academy of Sciences, Šlechtitelů 27, 78371 Olomouc, Czech Republic.

* Correspondence: wimmer@biomed.cas.cz or wimmerz@vscht.cz; Tel.: +420-241-062-457

1.1. Applied methods of conversion of 7 to 8, graphical image

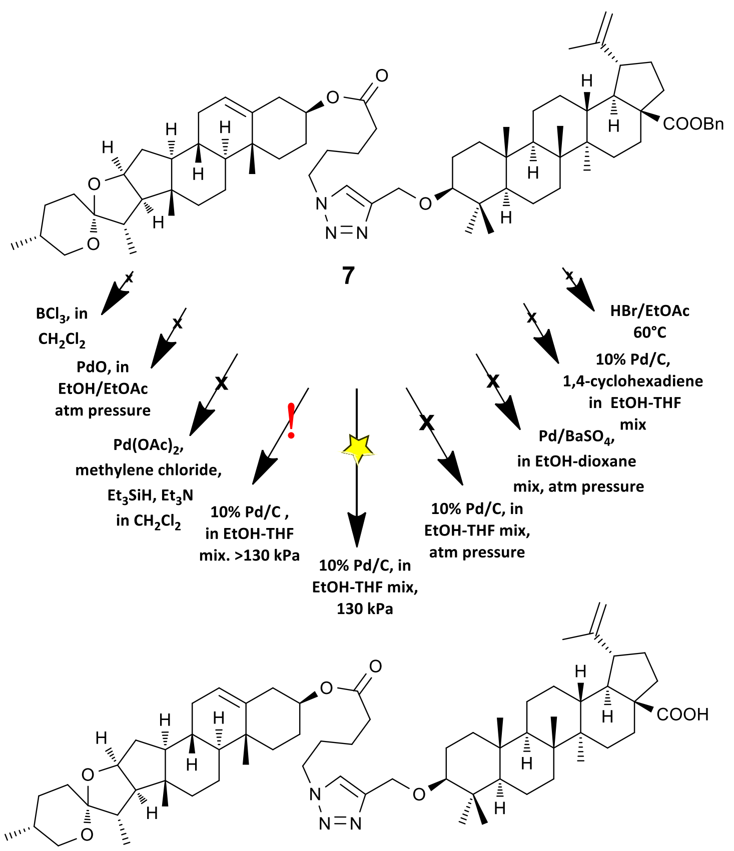

8

Scheme S1. Applied methods of conversion of **7** to **8**.

1.2. Physico-chemical and ADME characteristics

The partition coefficient (log *P*) and the distribution coefficient (log *D*) are the most important molecular descriptors [S1]. In chemical and pharmaceutical sciences, both, log *P* and log *D* are the measures of hydrophilicity or hydrophobicity of the studied compound, and are useful for estimating distribution of a drug within the body. The parameter log *D* shows the dependence on the pH of the matrix. Hydrophobic drugs are then preferentially distributed to hydrophobic compartments (e.g., lipid bilayer of cells), while hydrophilic drugs are preferentially distributed to hydrophilic compartments (e.g., blood serum). The distribution coefficient is a pH dependent value, and, therefore, the value at pH = 7.4 (the physiological

pH value of blood serum) is of particular importance (see also Table 2). Thus, log *P* expresses a ratio of concentrations of non-ionized compound between two phases, non-polar (octanol) and polar (water), while log *D* expresses the ratio of the sum of the concentrations of all forms of the compound (ionized and non-ionized) in each of the two phases. In pharmacology, log *P* and log *D* indicate how easily the drug can reach its intended target in the body, how strong its effect will be once it reaches its target and how long it will remain in the body in an active form. The log *P* values calculated for **1**, **2** and **5**–**8** exceed the values given by the Lipinski [28] and Ghose [29] rules (Table 4). Another supportive parameter is the predicted aqueous solubility, log *S*. The parameter *S* (in mol dm^{-3}) is the concentration of the solute in a saturated solution that is in equilibrium with the crystalline solid, and it is a pH dependent parameter. Standard range for log *S* at pH 7.4 is –6.5/+0.5. Among the studied compounds, only **1**, **2**, **5** and **6** show values for log *S* in this range (Table 4).

The importance of some of the ADME parameters for evaluation of pharmacokinetic properties of the prepared compounds is also summarized in Table 2. The blood brain barrier (BBB) and plasma protein binding are two of the important factors affecting distribution of the compound in the human body. Several parameters assist in evaluation of each potential drug for its BBB transport [S2]. The rate of brain penetration, log *PS*, is a logarithm of the permeability-surface area coefficient that measures the ability of a drug to cross the BBB and to move into brain tissue over time. It is one of the relevant parameters for evaluation of the rate of BBB penetration. The extent of brain penetration parameter, log *PB*, indicates if the drug might be active or inactive on the central nervous system (CNS). The typical values for log *PB* are –1.5/+1.5. Most of the current drugs show the log *PB* value up to +2 (active on CNS) or down to –2 (inactive on CNS). The log *PB* values calculated for **1**, **2** and **5**–**8** indicate that **1** is slightly active on CNS, while **2** and **5**–**8** are inactive on CNS (Table 2; Fig. S1 and Fig. S2). However, the complex of the BBB parameters is completed by the

brain/plasma equilibration rate, the parameter expressed as log *PS* * $f_{u,brain}$ that is a mathematical modeling parameter based on time required for reaching brain equilibrium. It is dependent on the brain unbound fraction ($f_{u,brain}$). This value indicates if the drug may potentially be active on CNS together with the log *BB* parameter, the predicted brain/blood partition coefficient. The parameter log *BB* is a hybrid parameter determined by permeability, plasma and brain tissue binding, and active transport mechanism, standard range –3.0/+1.2. The log *BB* values calculated for **1** and **5**–**8** appear in the required range of this parameter. Nevertheless, there are numerous exceptions already known.

Bioavailability represents another important ADME parameter. Among the studied compounds, the calculated bioavailability for **5**, **7** and **8** is lower than that for **1**, **2** and **6**. However, the experimental data show that only **5** and **7** showed cytotoxicity to the tested cancer cell lines.

Plasma protein binding (PPB) gives calculated quantity of a drug bound to a protein.

The Lipinski [28] and Ghose [29] rules describe molecular properties important for a small molecule drug pharmacokinetics in the human body, including their absorption, distribution, metabolism and excretion (ADME parameters). The importance of several ADME parameters, namely blood-brain barrier and plasma protein binding, reflects the distribution of the compound in the human body [S3]. A comparison of the calculated physico-chemical properties with the measured cytotoxicity shows that every time a new class of compound is being investigated, no available experimental screening may be intentionally omitted (Tables 3 and 4).

1.3. Calculated activity on central nervous system (CNS)

Fig. S1 shows the calculated activity of diosgenin (**1**) and betulinic acid (**2**) on central nervous system. Blue dots represent currently used drugs that are CNS active, while orange dots

represent currently used drugs that are CNS inactive. The green dot shows the calculated position of diosgenin (**1**). The calculation was made through the ACD/Labs software [27]. In the below shown graph, log PS * $f_{u,brain}$ (the brain/plasma equilibration rate) appears on the x-axis, and log BB (a hybrid parameter determined by permeability, plasma and brain tissue binding, and active transport mechanism) appears on the y-axis. Based on the position of the green dot, diosgenin (**1**) displays certain potential to become active on central nervous system, which result of the calculation is in good agreement that **1** was determined as adaptogen.

In turn, the compounds **5** and **7** appeared to have no CNS activity at all, as shown in the Fig. S2.

Figure S1. A comparison of the calculated position of diosgenin (**1**; green dot) and betulinic acid (**2**; green dot) in the diagram of the currently used CNS active drugs (blue dots) and CNS inactive drugs (orange dots).

Diosgenin (**1**)

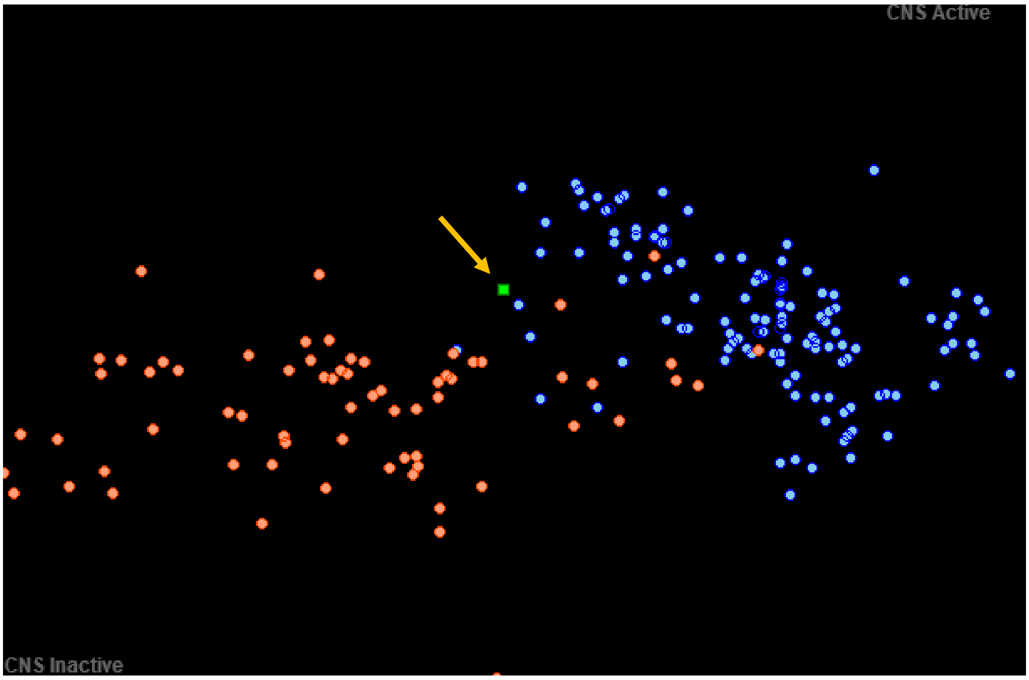

Betulinic acid (**2**)

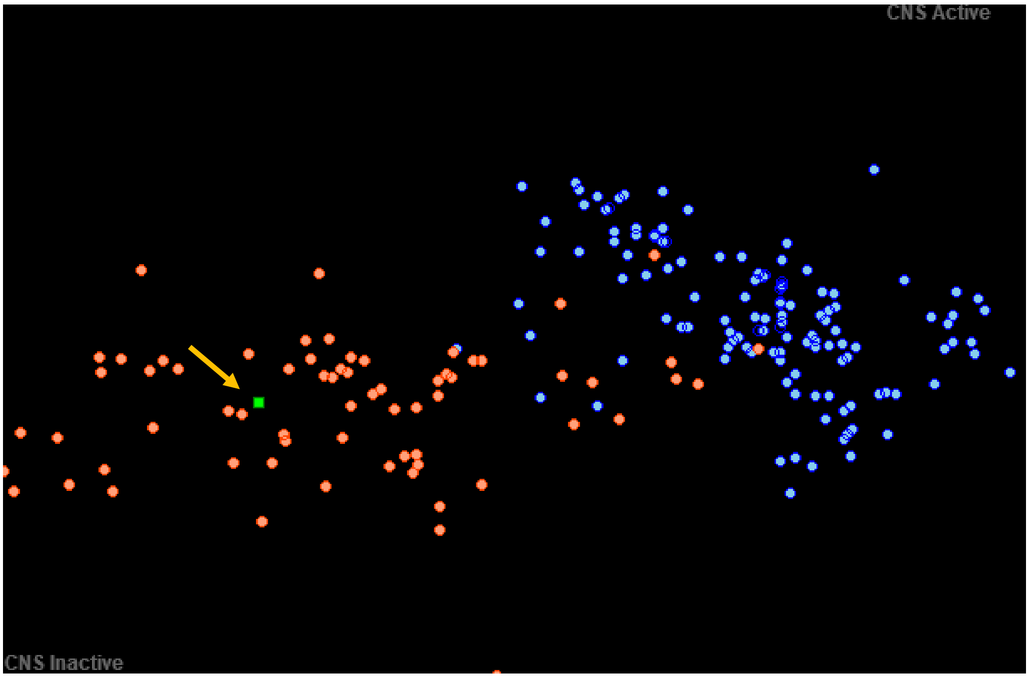

Figure S2. Calculated CNS activity of **5** (A), **6** (B), **7** (C) and **8** (D). Green dots are located in the left bottom corners of the pictures (for A, C and D).

A

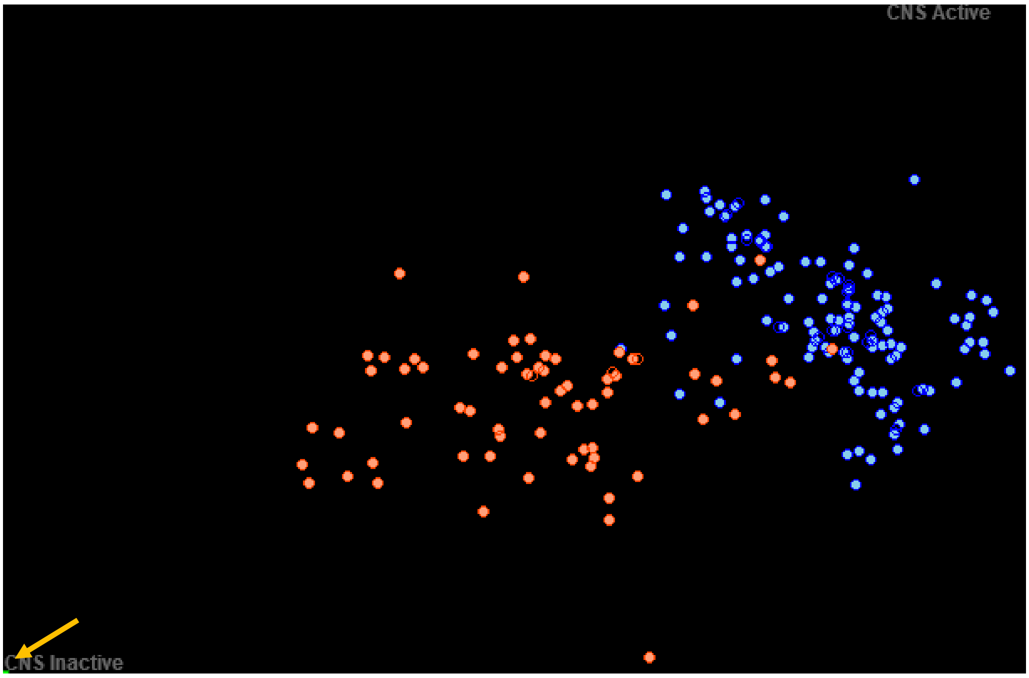

B

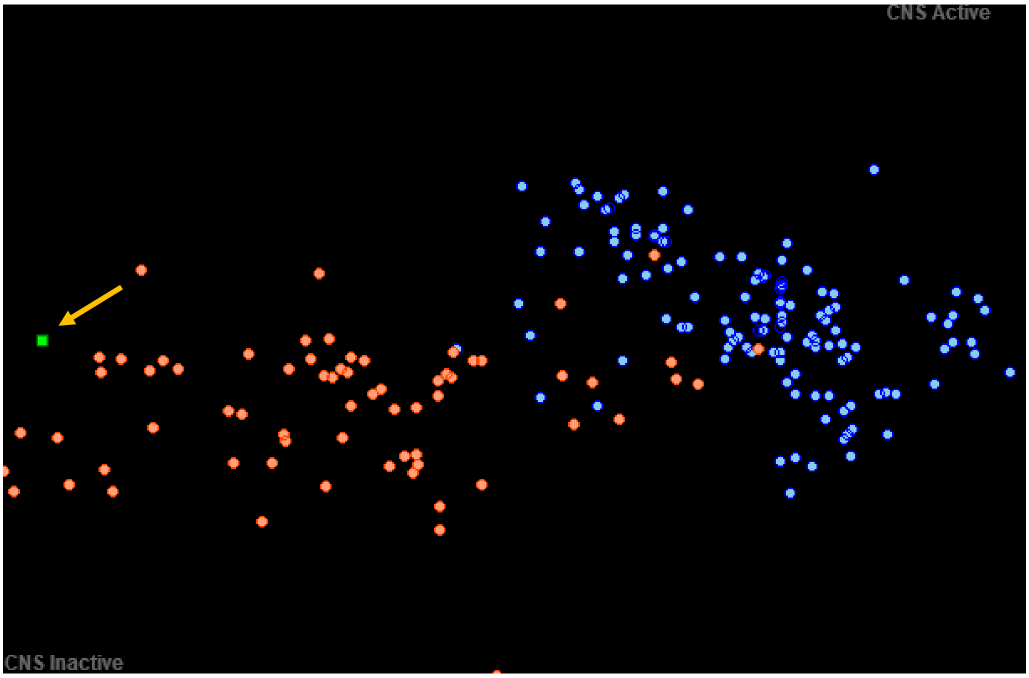

C

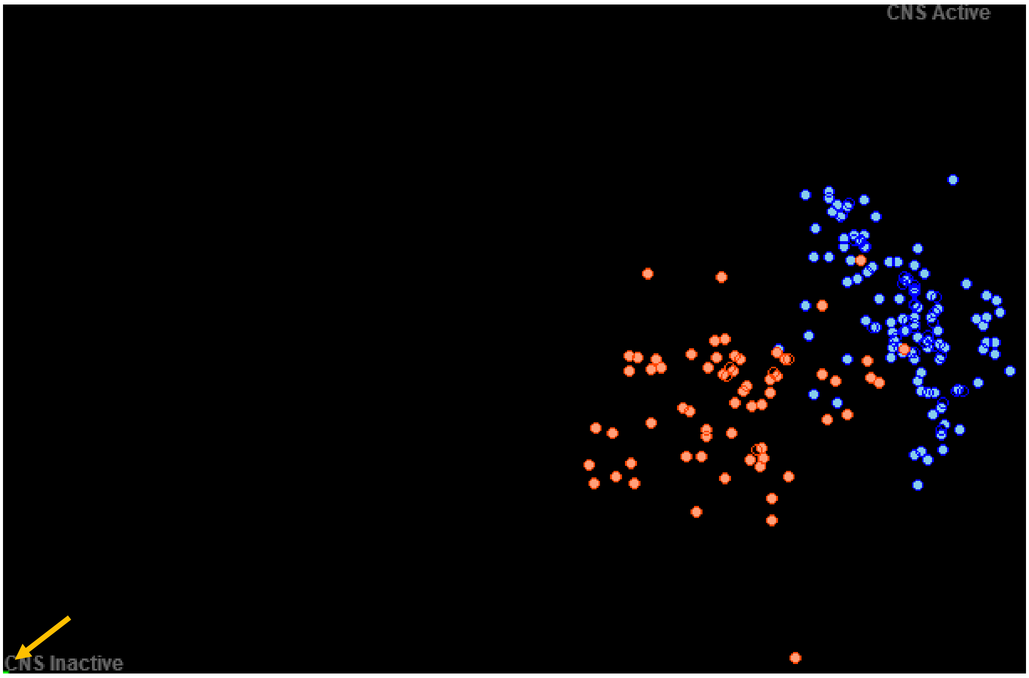

D

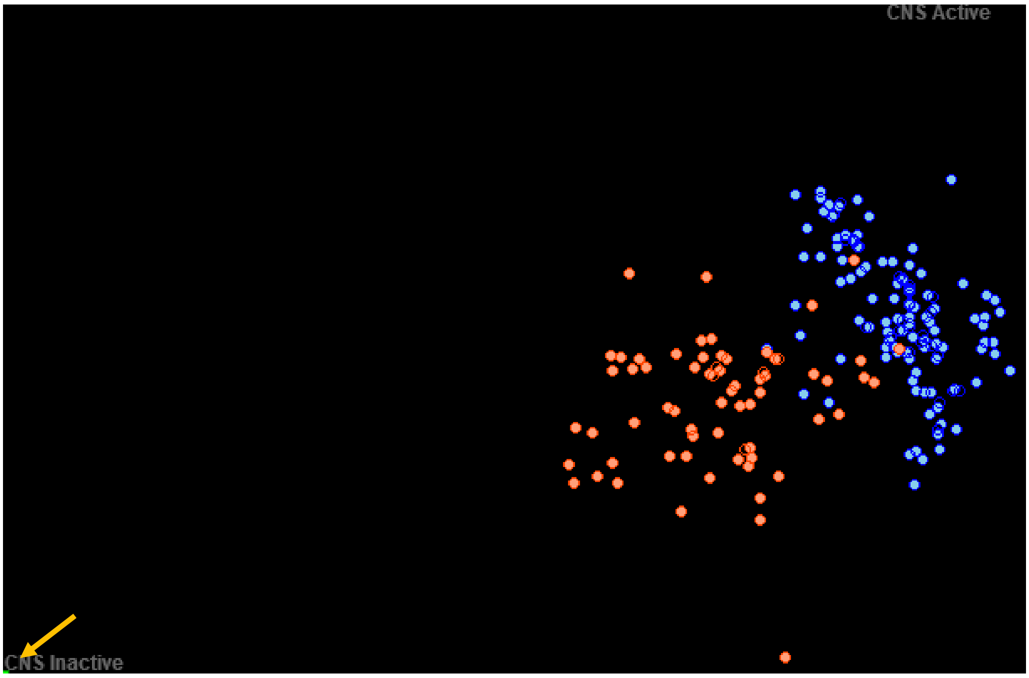

1.4. References

[S1] Kujawski, J.; Popielarska, H.; Myka, A.; Drabinska, B.; Bernard, M.K. The log *P* parameter as a molecular descriptor in the computer-aided drug design – an overview. *Comp. Methods Sci. Technol.* **2012**, *18*, 81–88.

[S2] Zhao, R.; Kalvass, J.C.; Pollack, G.M. Assessment of blood–brain barrier permeability using the in situ mouse brain perfusion technique. *Pharm. Res.* **2009**, *26*, 1657–1664.

[S3] Kalani, K.; Yadav, D.K.; Khan, F.; Srivastava, S.K.; N. Suri, N. Pharmacophore, QSAR, and ADME based semisynthesis and *in vitro* evaluation of ursolic acid analogs for anticancer activity. *J. Mol. Model.* **2012**, *18*, 3389–3413.

1.5. NMR spectra of the prepared compounds

Compound **3**

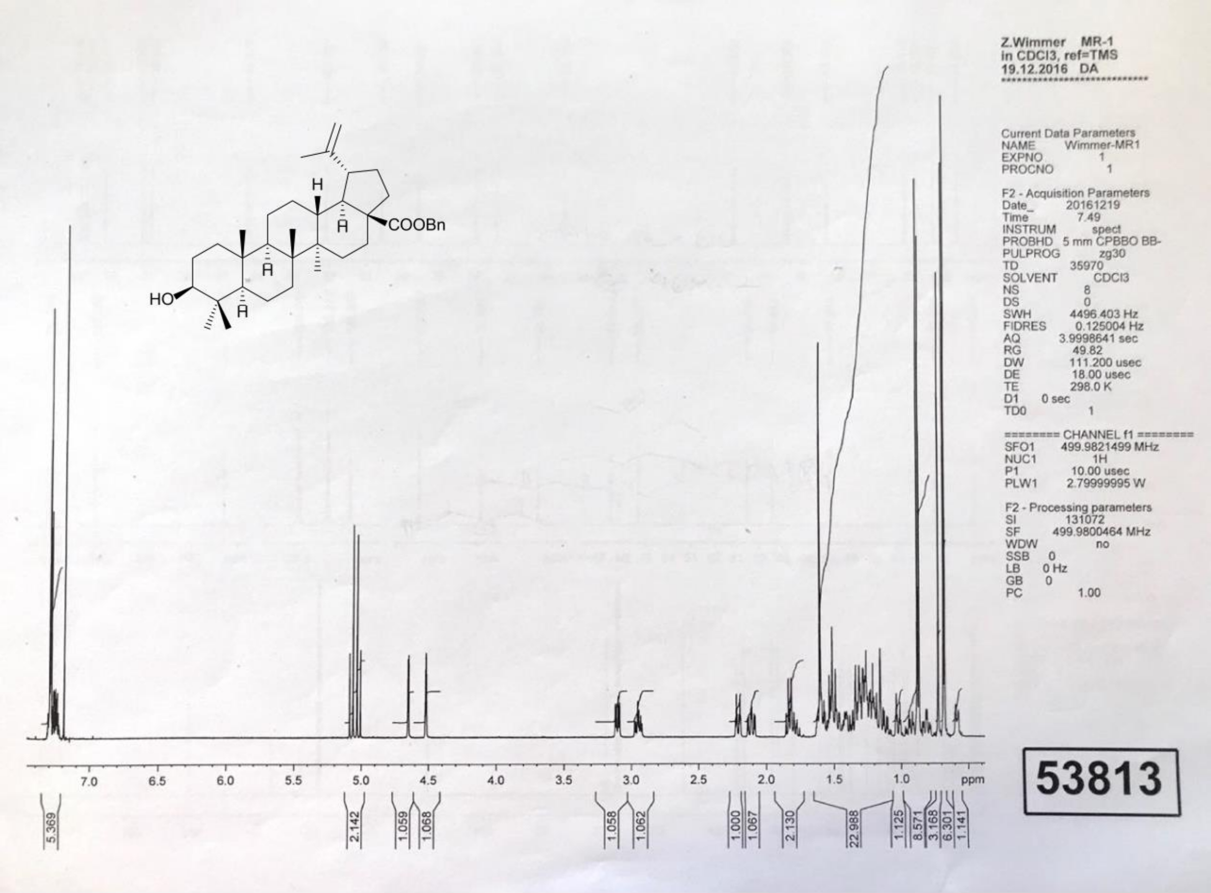

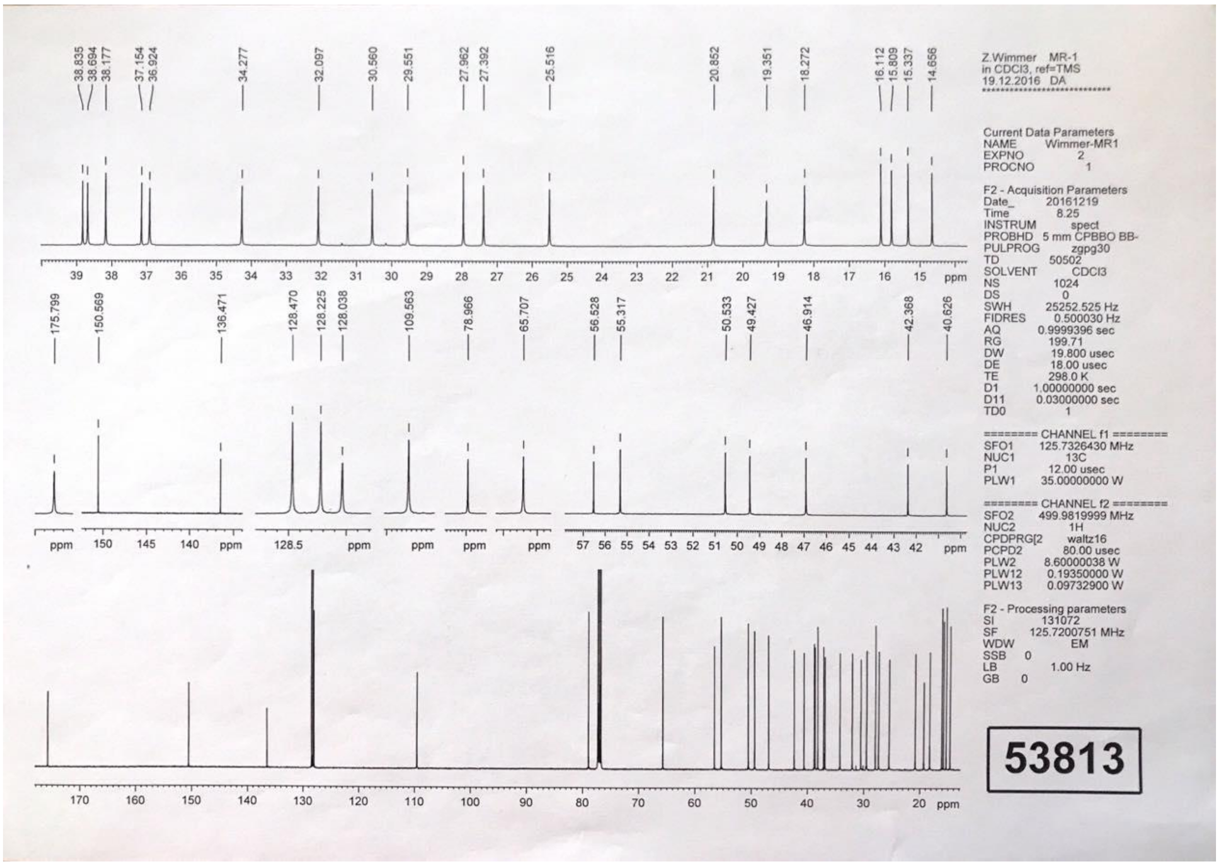
Z.Wimmer MR-1
in CDCl3, ref=TMS
19.12.2016 DA
Current Data Parameters
NAME Wimmer-MR1
EXPNO 2
PROCNO 1
53813

Compound **4**

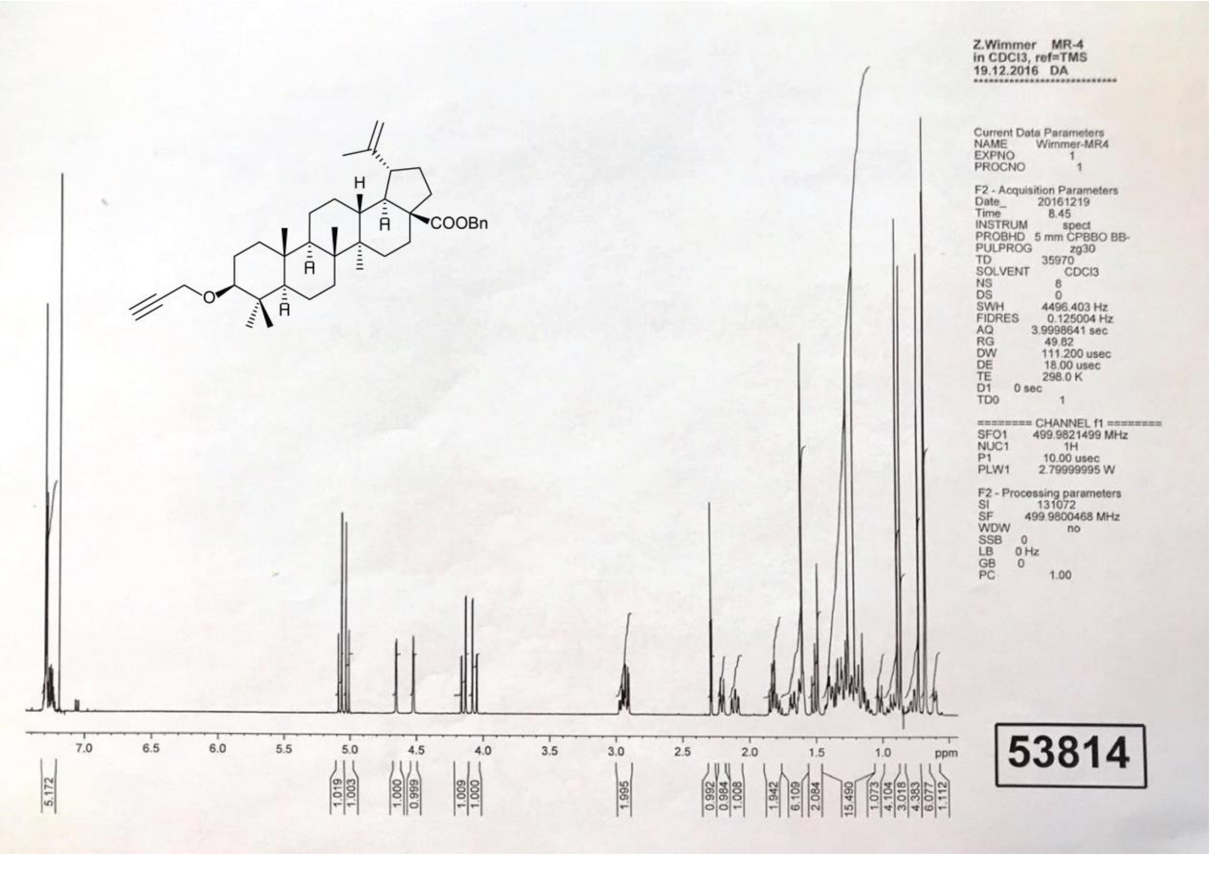
Z.Wimmer MR-4
in CDCl3, ref=TMS
19.12.2016 DA
COOBn
Current Data Parameters
NAME Wimmer-MR4
EXPNO 1
PROCNO 1
53814

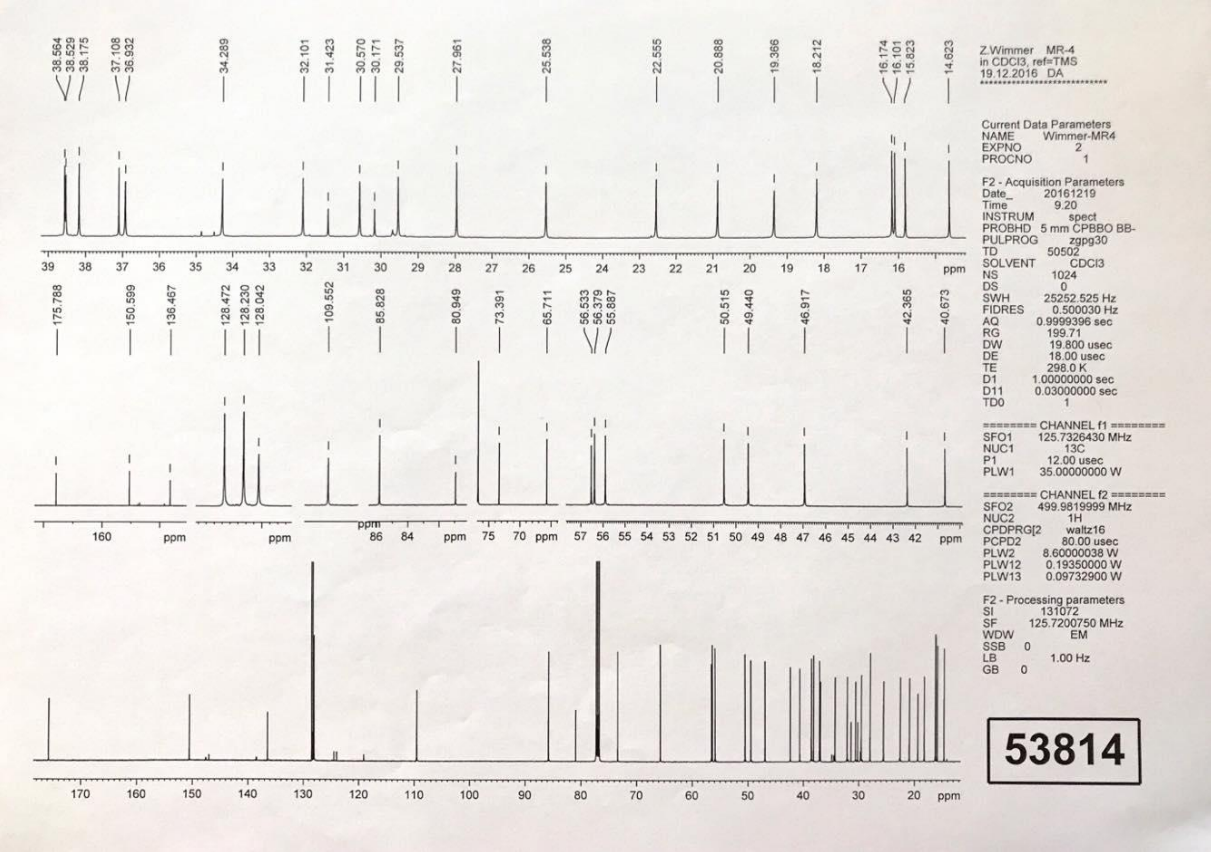
Z.Wimmer MR-4
in CDCl3, ref=TMS
19.12.2016 DA
Current Data Parameters
NAME Wimmer-MR4
EXPNO 2
PROCNO 1
F2 - Acquisition Parameters
Date_ 20161219
Time 9.20
INSTRUM spect
PROBHD 5 mm CPBBO BB-
PULPROG zgpg30
TD 50502
SOLVENT CDCl3
NS 1024
DS 0
SWH 25252.525 Hz
FIDRES 0.500030 Hz
AQ 0.9999396 sec
RG 199.71
DW 19.800 usec
DE 18.00 usec
TE 298.0 K
D1 1.00000000 sec
D11 0.03000000 sec
TD0 1
======== CHANNEL f1 ========
SFO1 125.7326430 MHz
NUC1 13C
P1 12.00 usec
PLW1 35.00000000 W
======== CHANNEL f2 ========
SFO2 499.9819999 MHz
NUC2 1H
CPDPRG[2 waltz16
PCPD2 80.00 usec
PLW2 8.60000038 W
PLW12 0.19350000 W
PLW13 0.09732900 W
F2 - Processing parameters
SI 131072
SF 125.7200750 MHz
WDW EM
SSB 0
LB 1.00 Hz
GB 0
53814

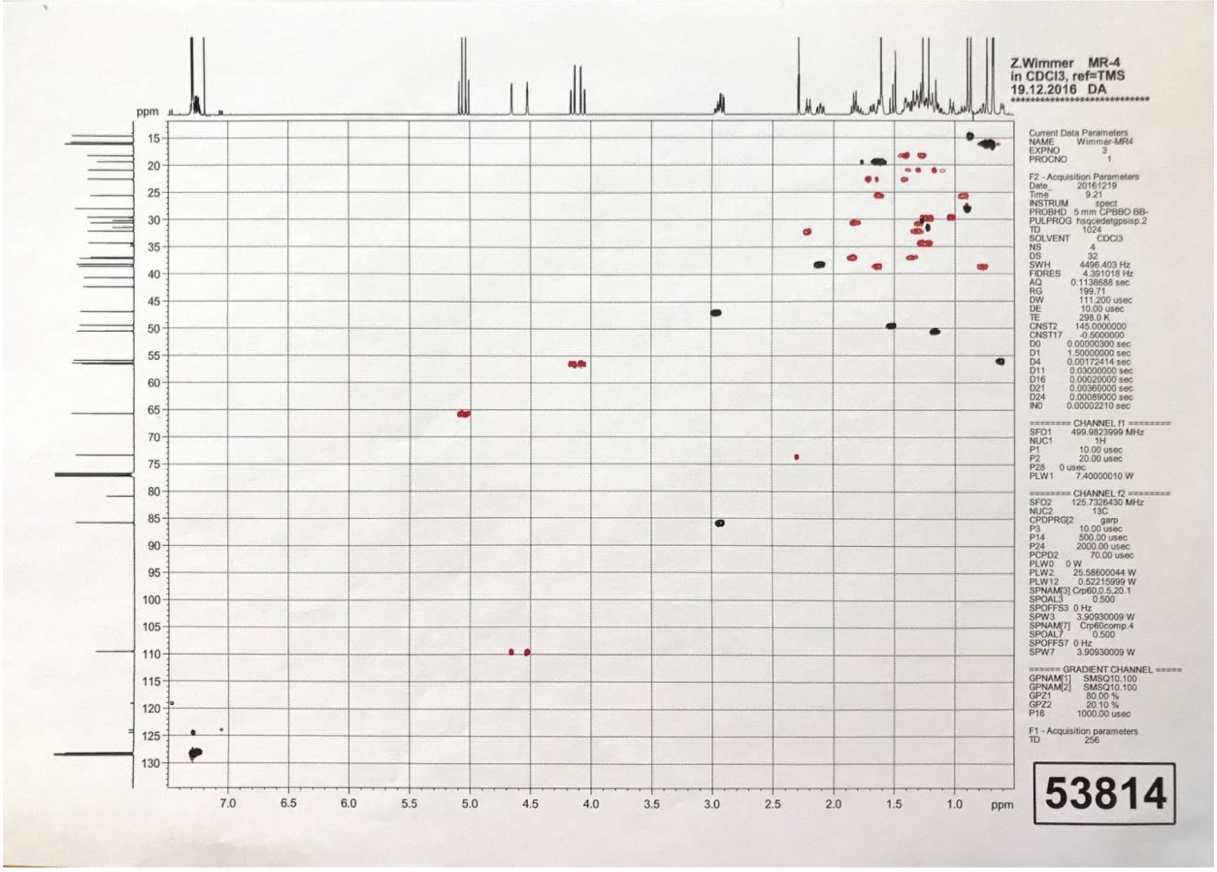
Z.Wimmer MR-4
in CDCl3, ref=TMS
19.12.2016 DA
Current Data Parameters
NAME Wimmer-MR4
EXPNO 3
PROCNO 1
F2 - Acquisition Parameters
Date_ 20161219
Time 9.21
INSTRUM spect
PROBHD 5 mm CPBBO BB-
PULPROG hsqcedetgpsisp.2
TD 1024
SOLVENT CDCl3
NS 4
DS 32
SWH 4496.403 Hz
FIDRES 4.391018 Hz
AQ 0.1138688 sec
RG 199.71
DW 111.200 usec
DE 10.00 usec
TE 298.0 K
======== CHANNEL f1 ========
SFO1 499.9823999 MHz
NUC1 1H
======== CHANNEL f2 ========
SFO2 125.7326430 MHz
NUC2 13C
CPDPRG[2 garp
====== GRADIENT CHANNEL =====
F1 - Acquisition parameters
TD 256
53814

Compound **5**

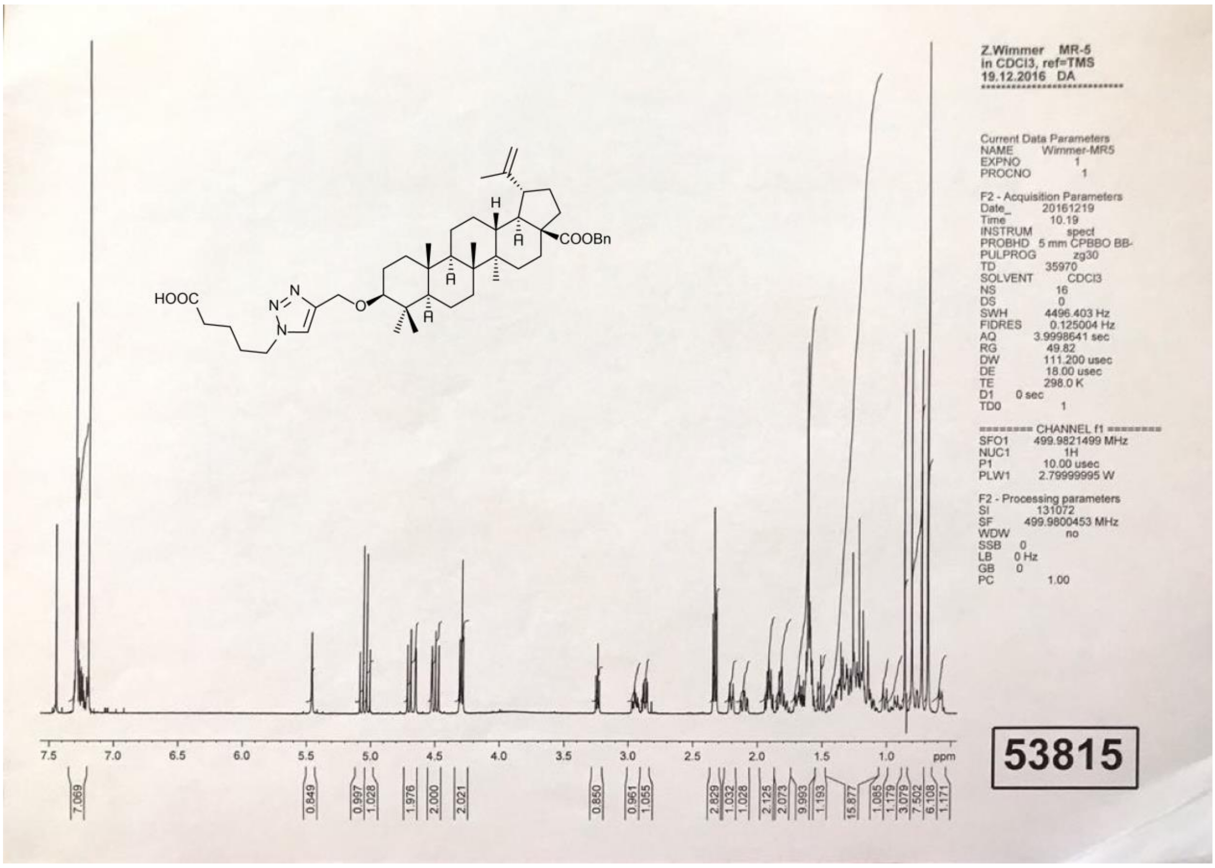
Z.Wimmer MR-5
in CDCl3, ref=TMS
19.12.2016 DA
53815

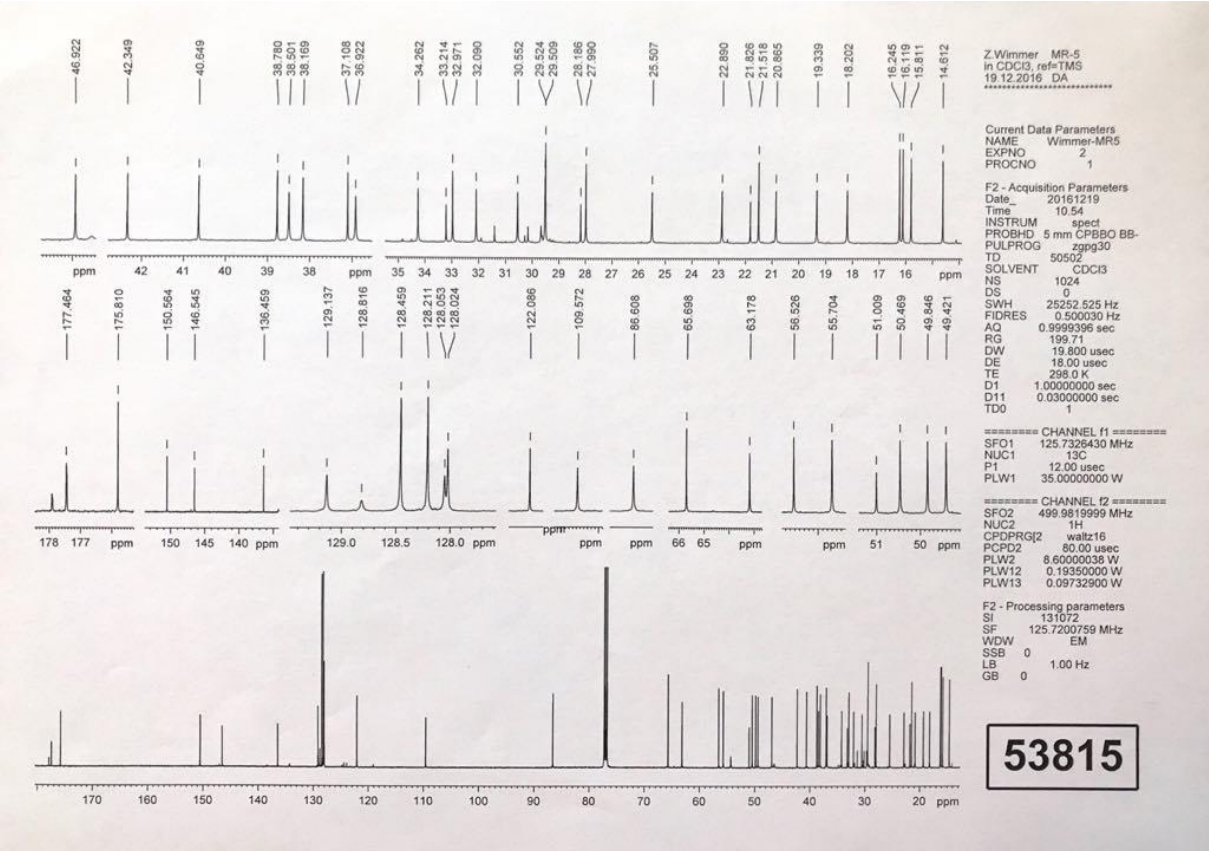
Z.Wimmer MR-5
in CDCl3, ref=TMS
19.12.2016 DA
53815

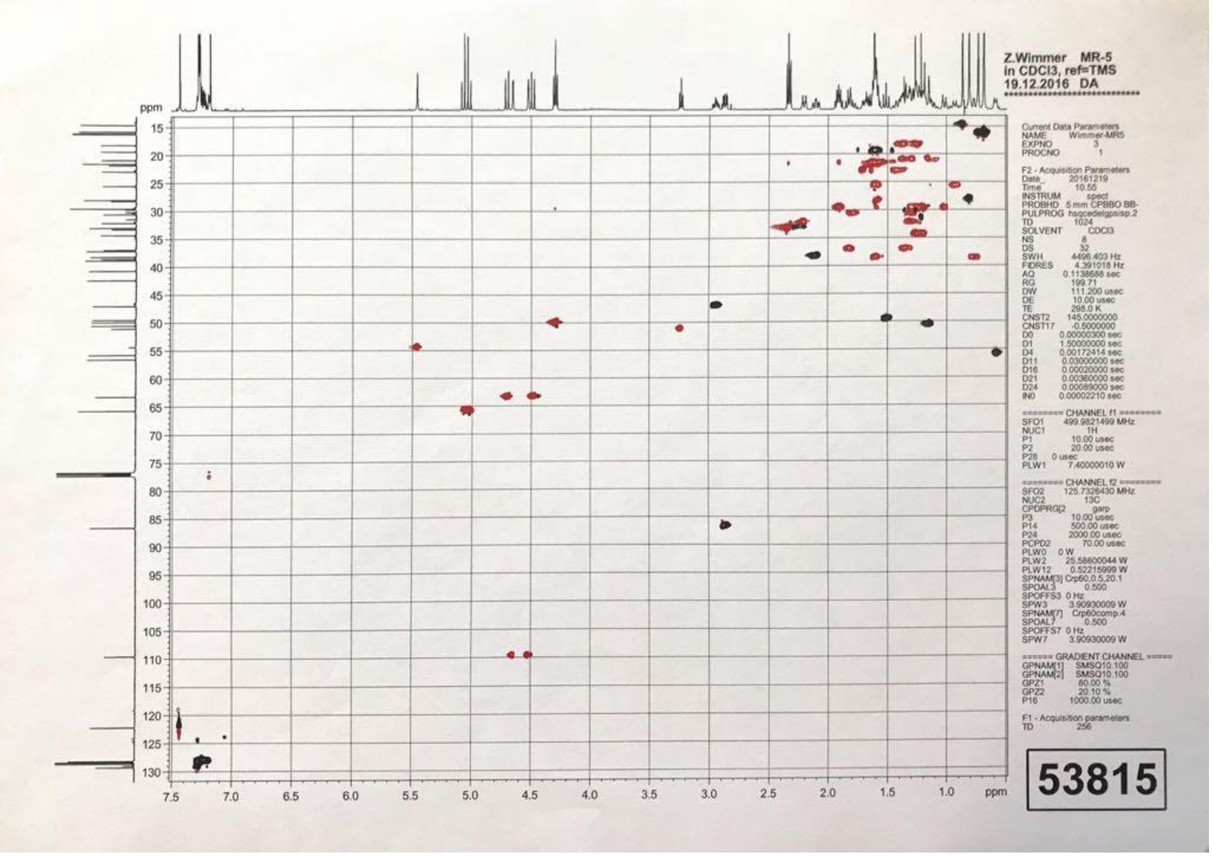

Compound **6**

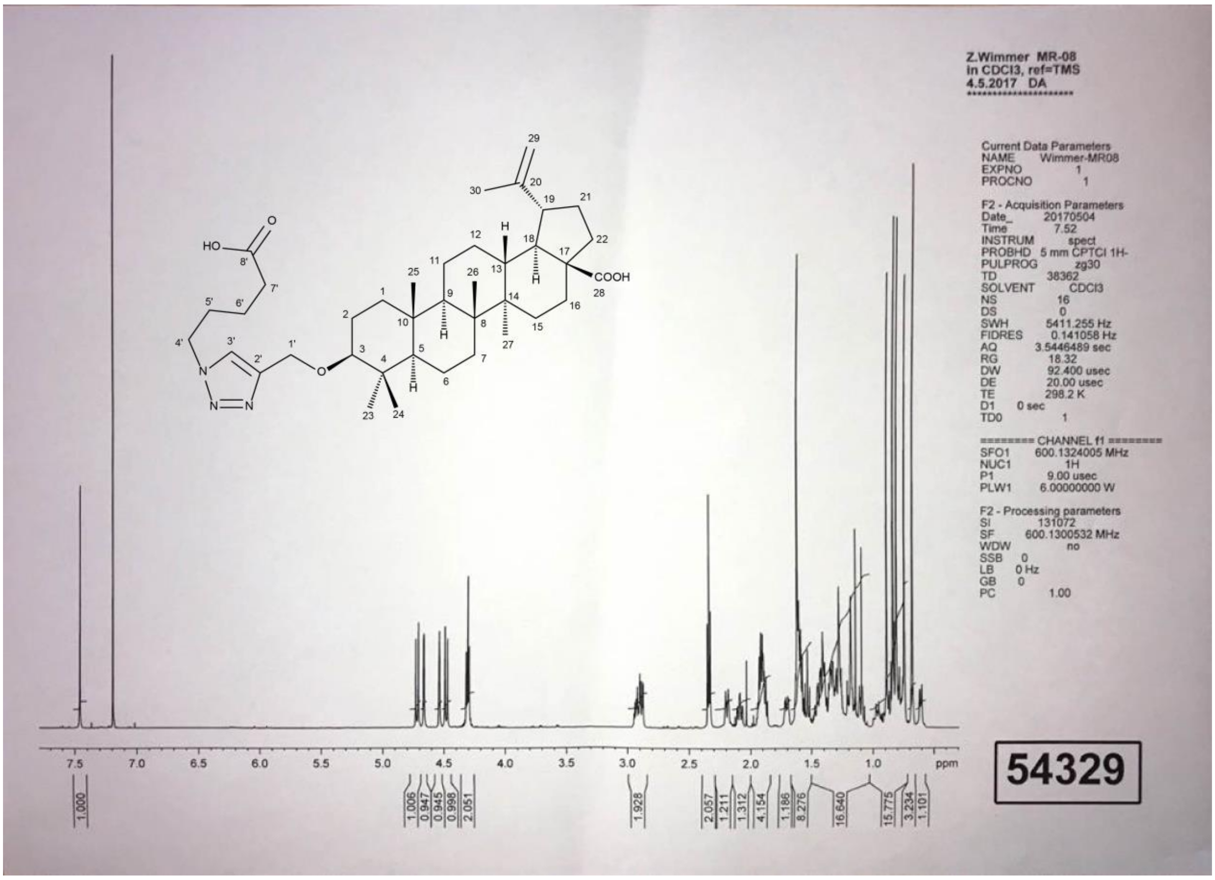

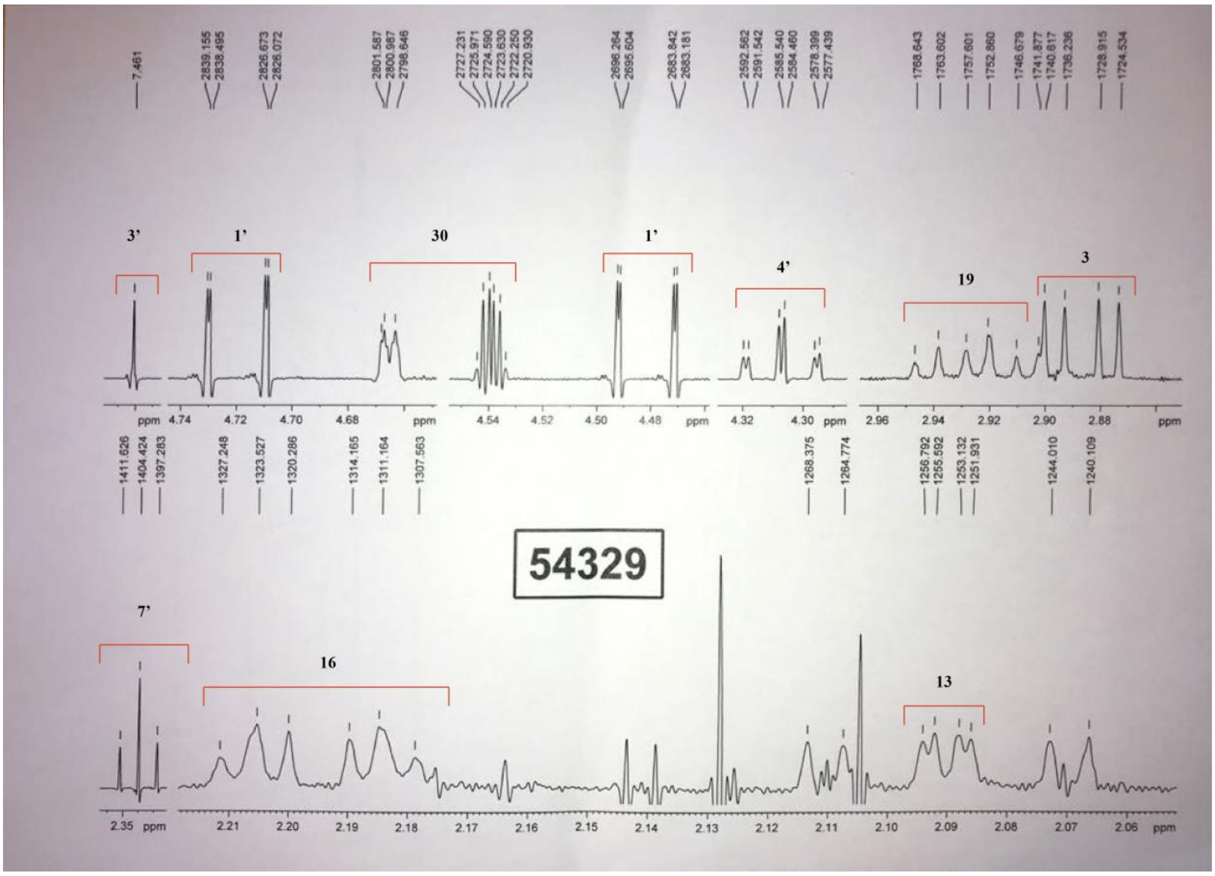
3'
1'
30
1'
4'
19
3
54329
7'
16
13

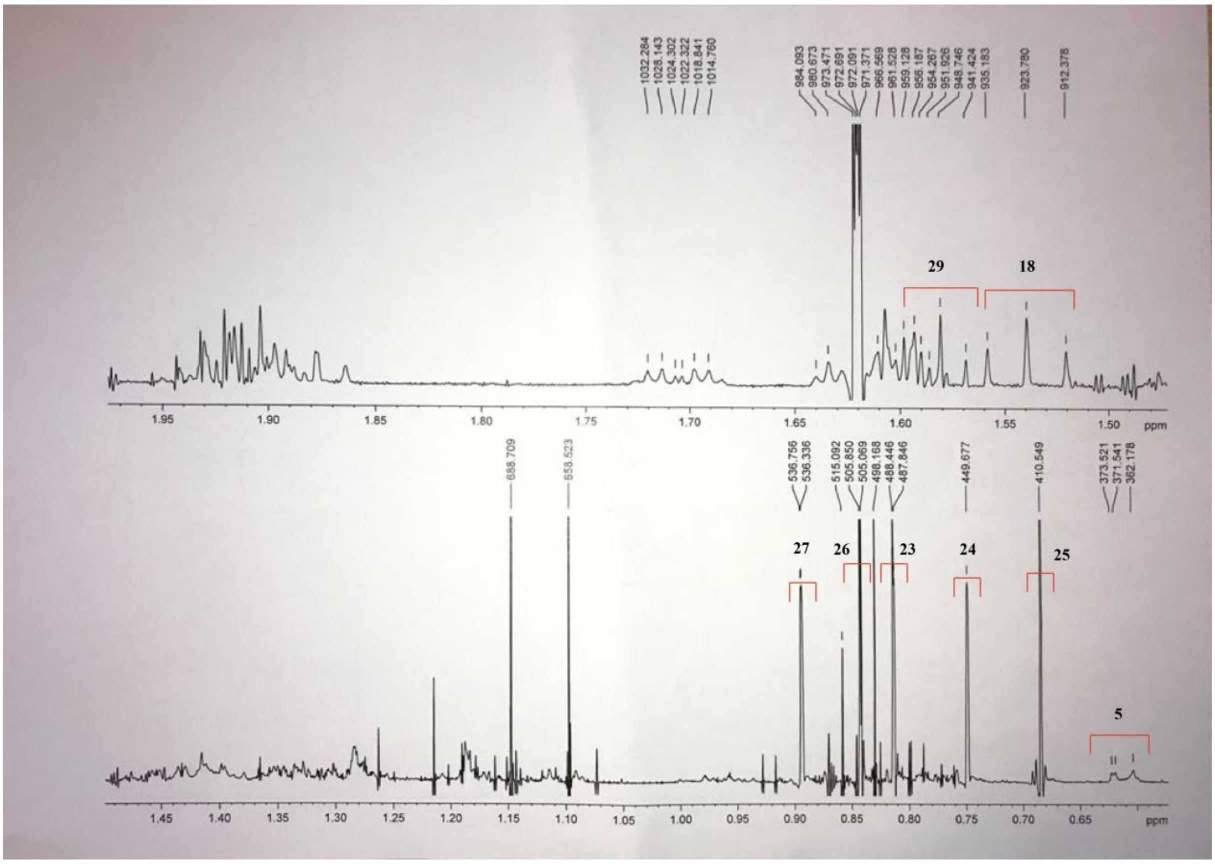
29
18
27
26
23
24
25
5

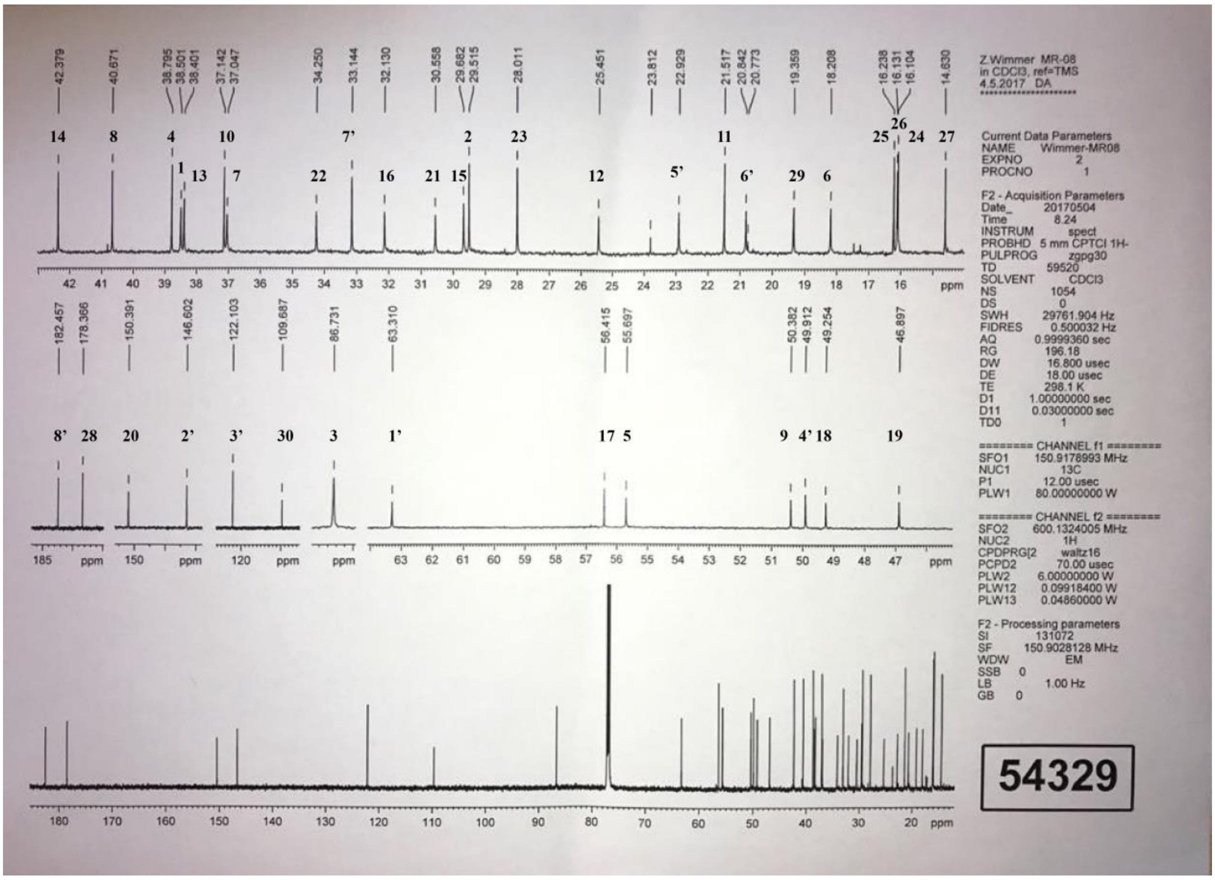

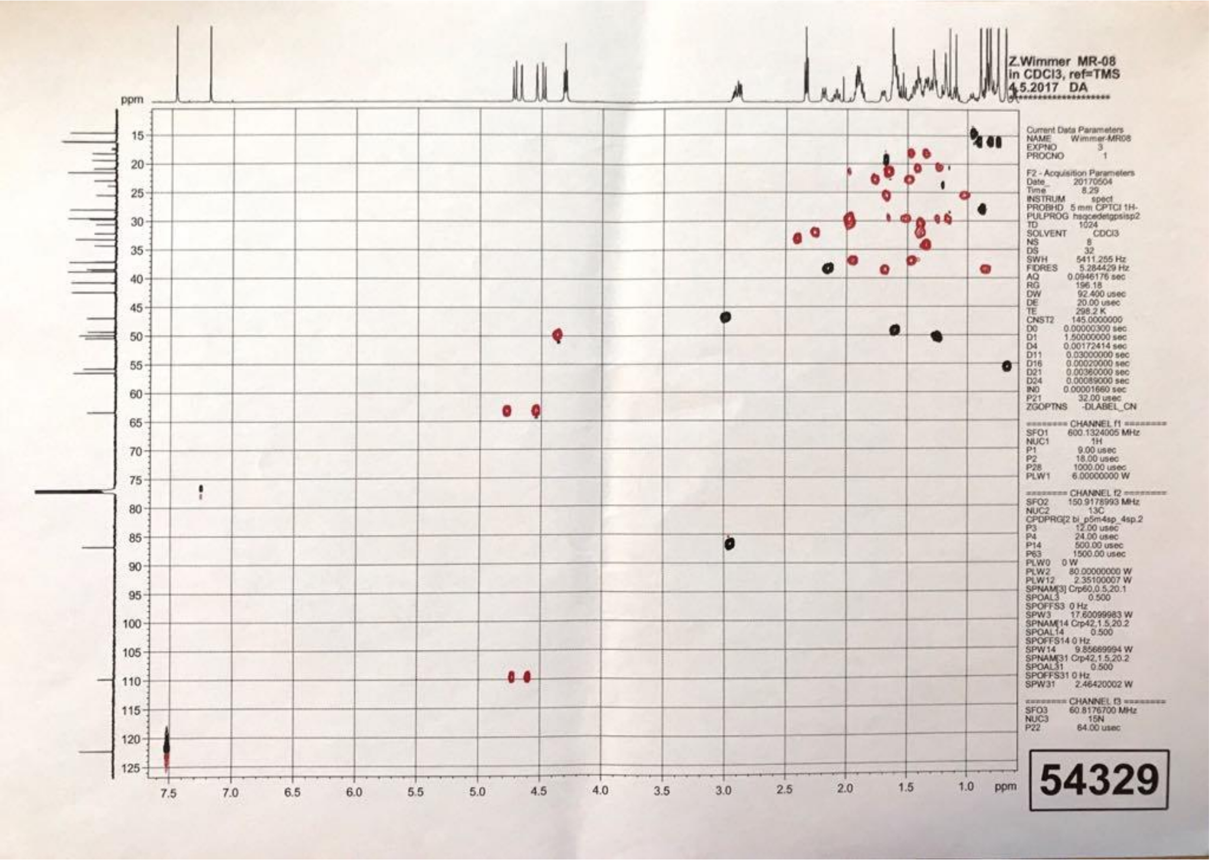

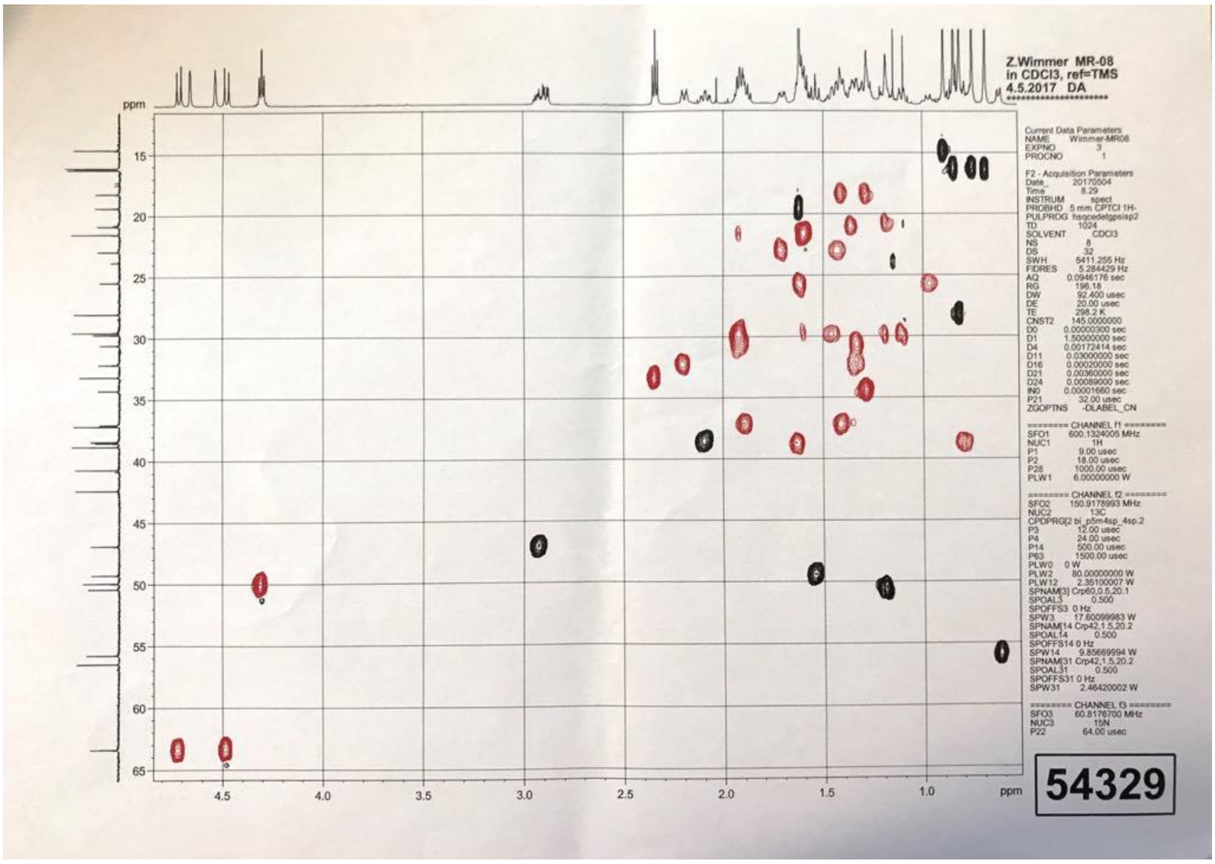
Z.Wimmer MR-08
in CDCl3, ref=TMS
4.5.2017 DA
54329

Compound **7**

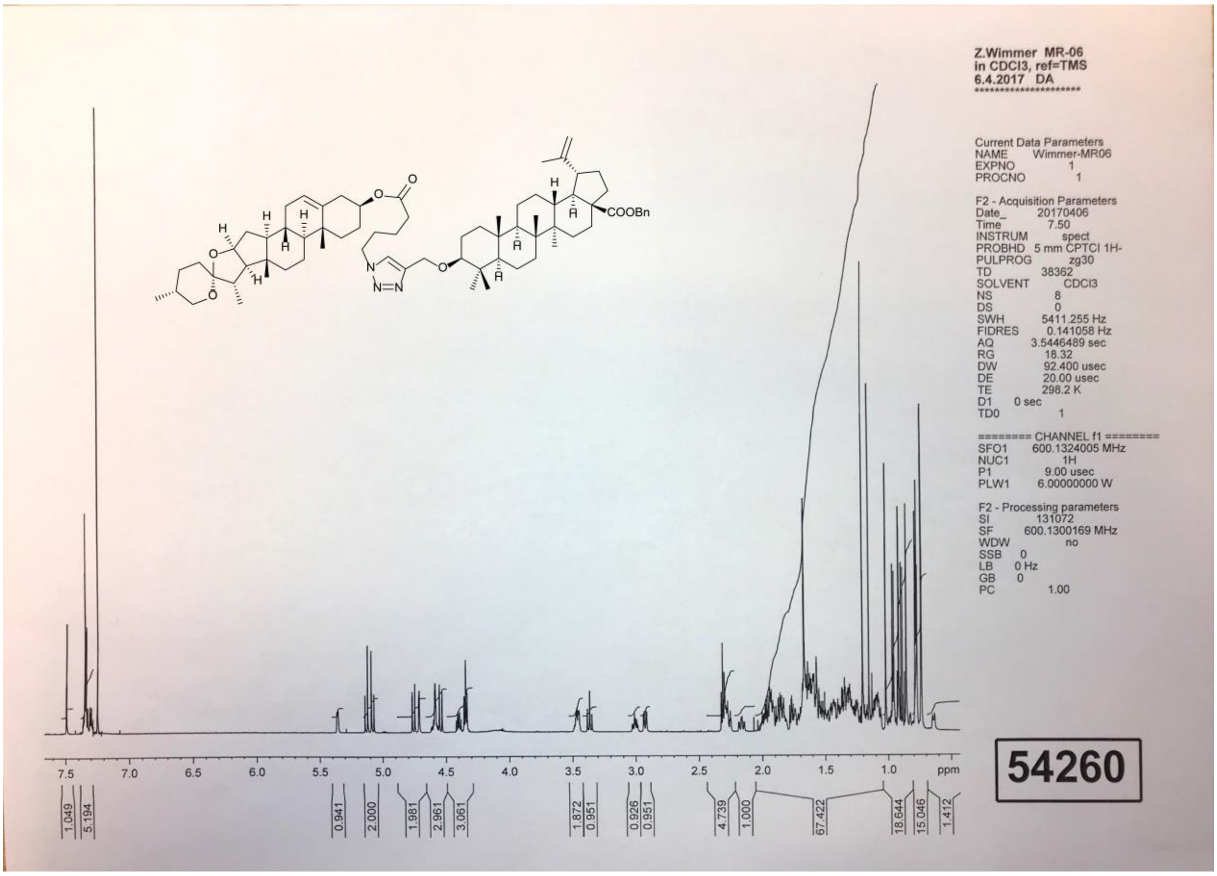
Z.Wimmer MR-06
in CDCl3, ref=TMS
6.4.2017 DA
COOBn
54260

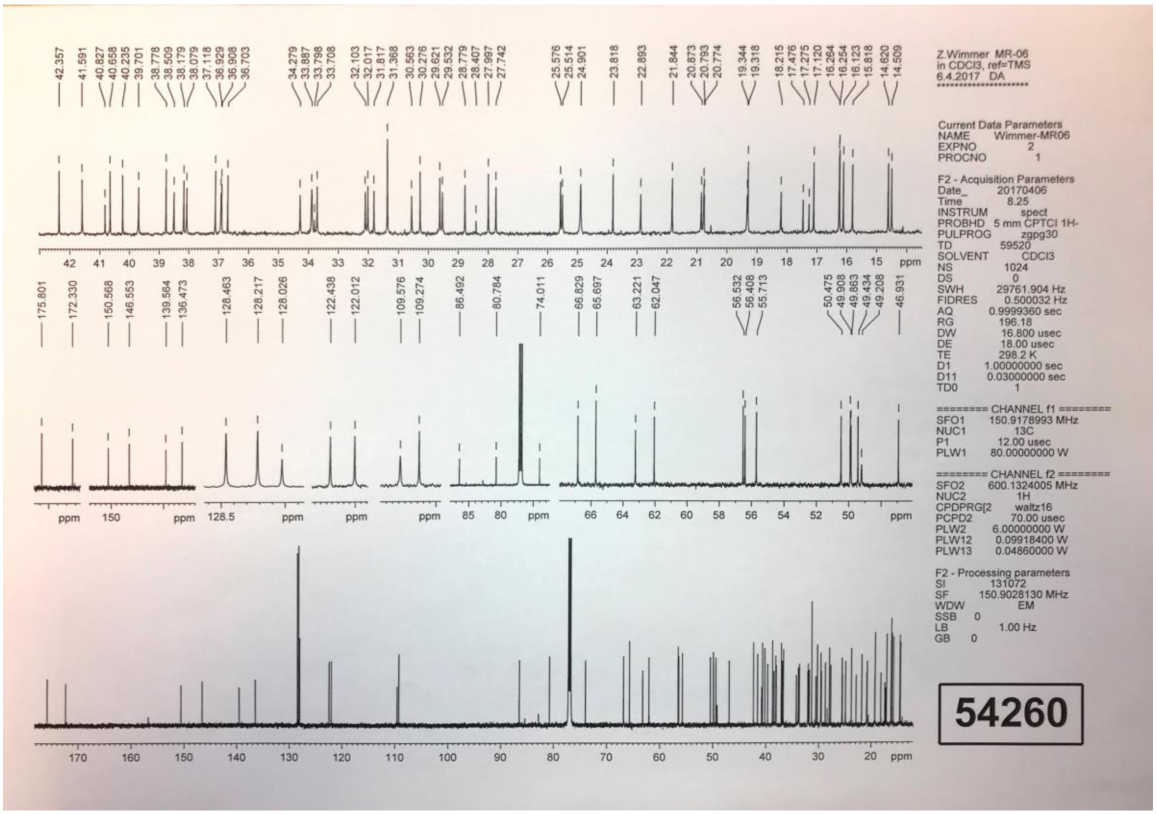
Z.Wimmer MR-06
in CDCl3, ref=TMS
6.4.2017 DA
Current Data Parameters
NAME Wimmer-MR06
EXPNO 2
PROCNO 1
F2 - Acquisition Parameters
Date_ 20170406
Time 8.25
INSTRUM spect
PROBHD 5 mm CPTCI 1H-
PULPROG zgpg30
TD 59520
SOLVENT CDCl3
NS 1024
DS 0
SWH 29761.904 Hz
FIDRES 0.500032 Hz
AQ 0.9999360 sec
RG 196.18
DW 16.800 usec
DE 18.00 usec
TE 298.2 K
D1 1.00000000 sec
D11 0.03000000 sec
TD0 1
======== CHANNEL f1 ========
SFO1 150.9178993 MHz
NUC1 13C
P1 12.00 usec
PLW1 80.00000000 W
======== CHANNEL f2 ========
SFO2 600.1324005 MHz
NUC2 1H
CPDPRG[2 waltz16
PCPD2 70.00 usec
PLW2 6.00000000 W
PLW12 0.09918400 W
PLW13 0.04860000 W
F2 - Processing parameters
SI 131072
SF 150.9028130 MHz
WDW EM
SSB 0
LB 1.00 Hz
GB 0
54260

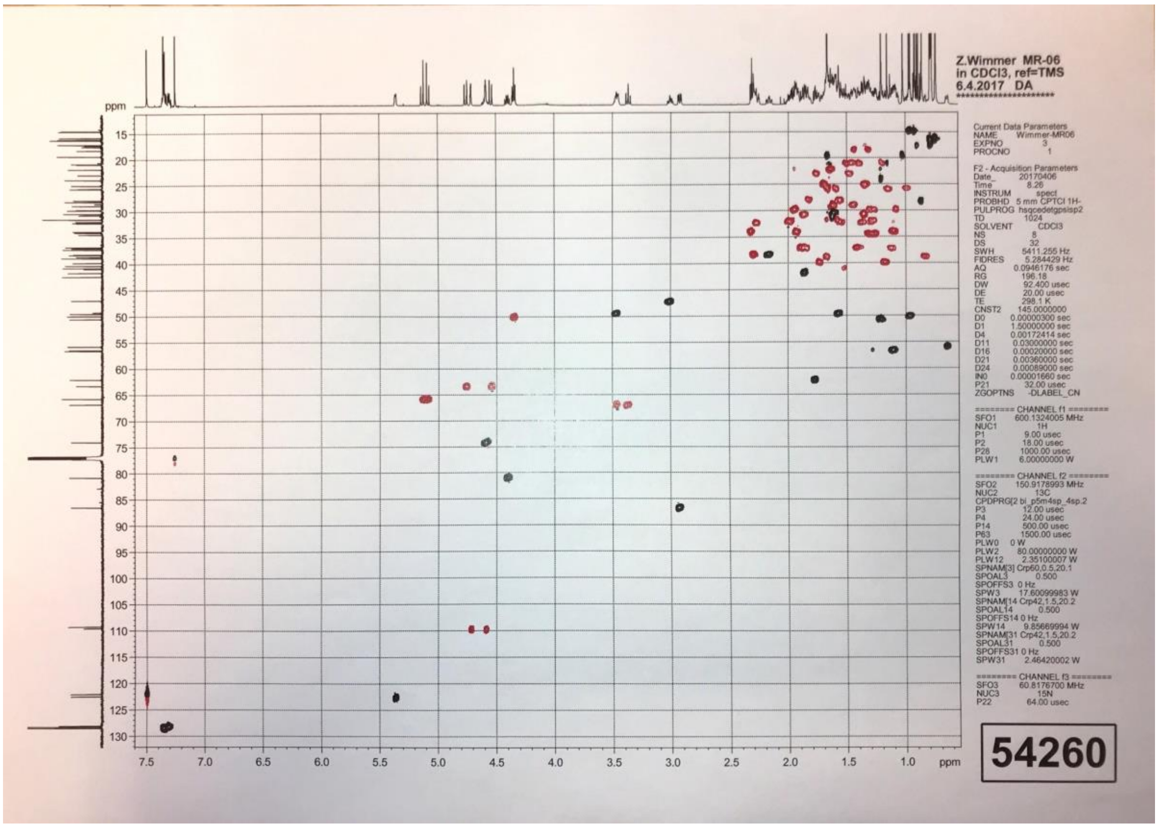
Z.Wimmer MR-06
in CDCl3, ref=TMS
6.4.2017 DA
Current Data Parameters
NAME Wimmer-MR06
EXPNO 3
PROCNO 1
54260

Compound **8**

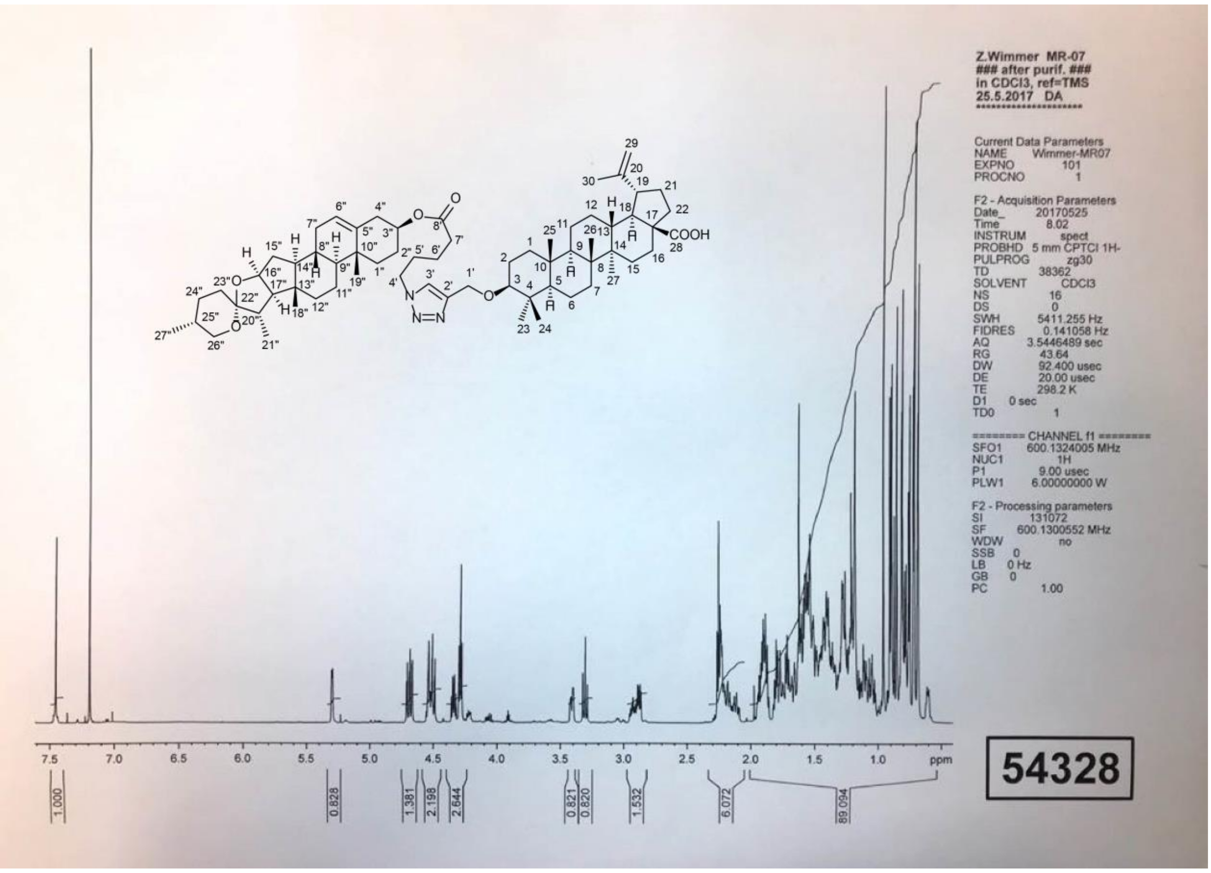

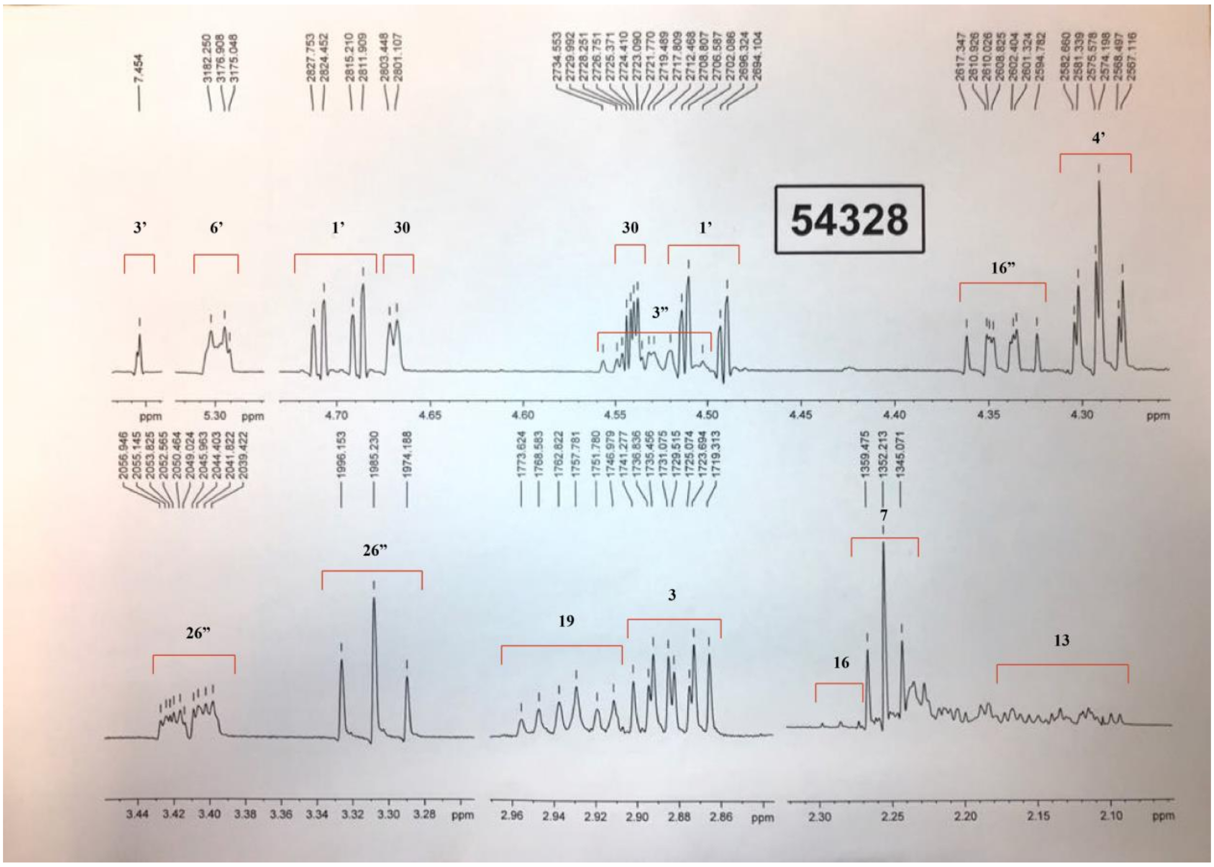

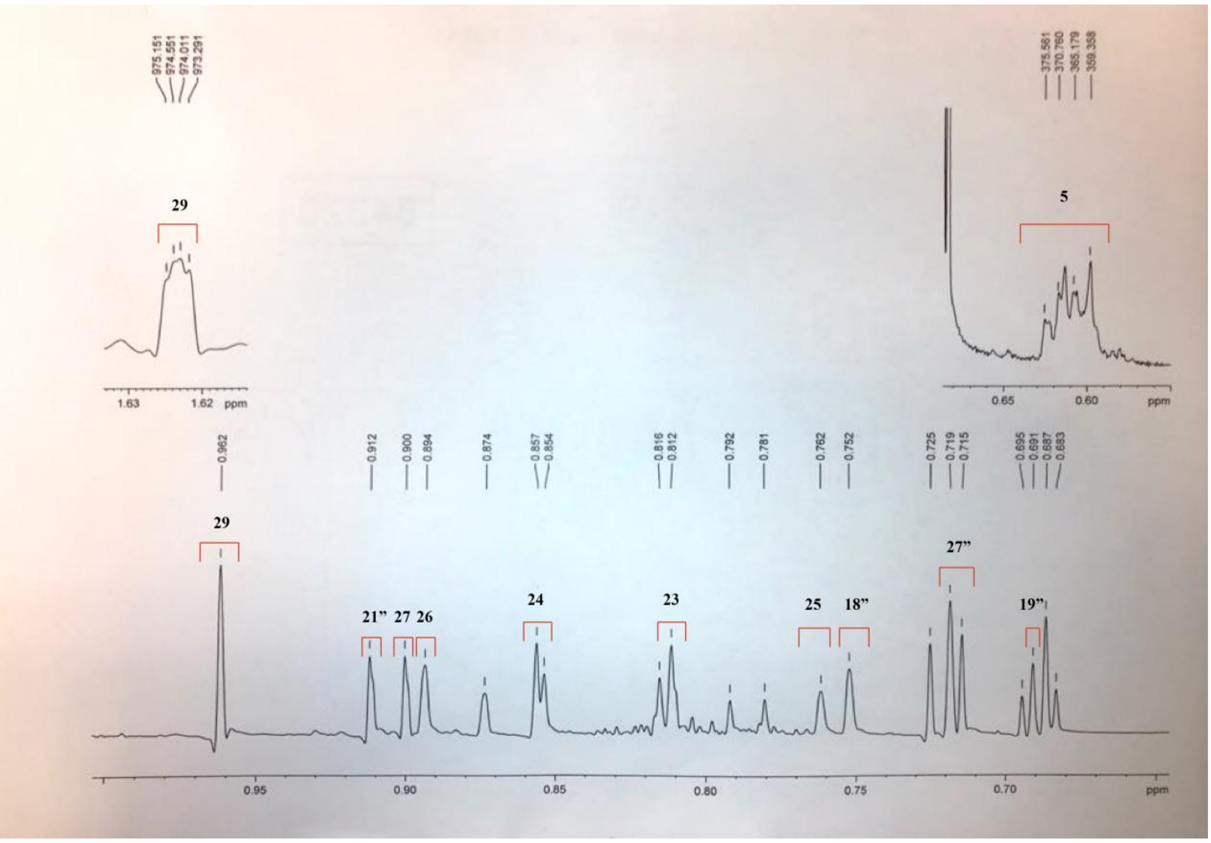
975.151
974.551
974.011
973.291
29
1.63
1.62 ppm
375.561
370.760
365.179
359.358
5
0.65
0.60
ppm
0.962
0.912
0.900
0.894
0.874
0.857
0.854
0.816
0.812
0.792
0.781
0.762
0.752
0.725
0.719
0.715
0.695
0.691
0.687
0.683
29
21” 27 26
24
23
25 18”
27”
19”
0.95
0.90
0.85
0.80
0.75
0.70
ppm

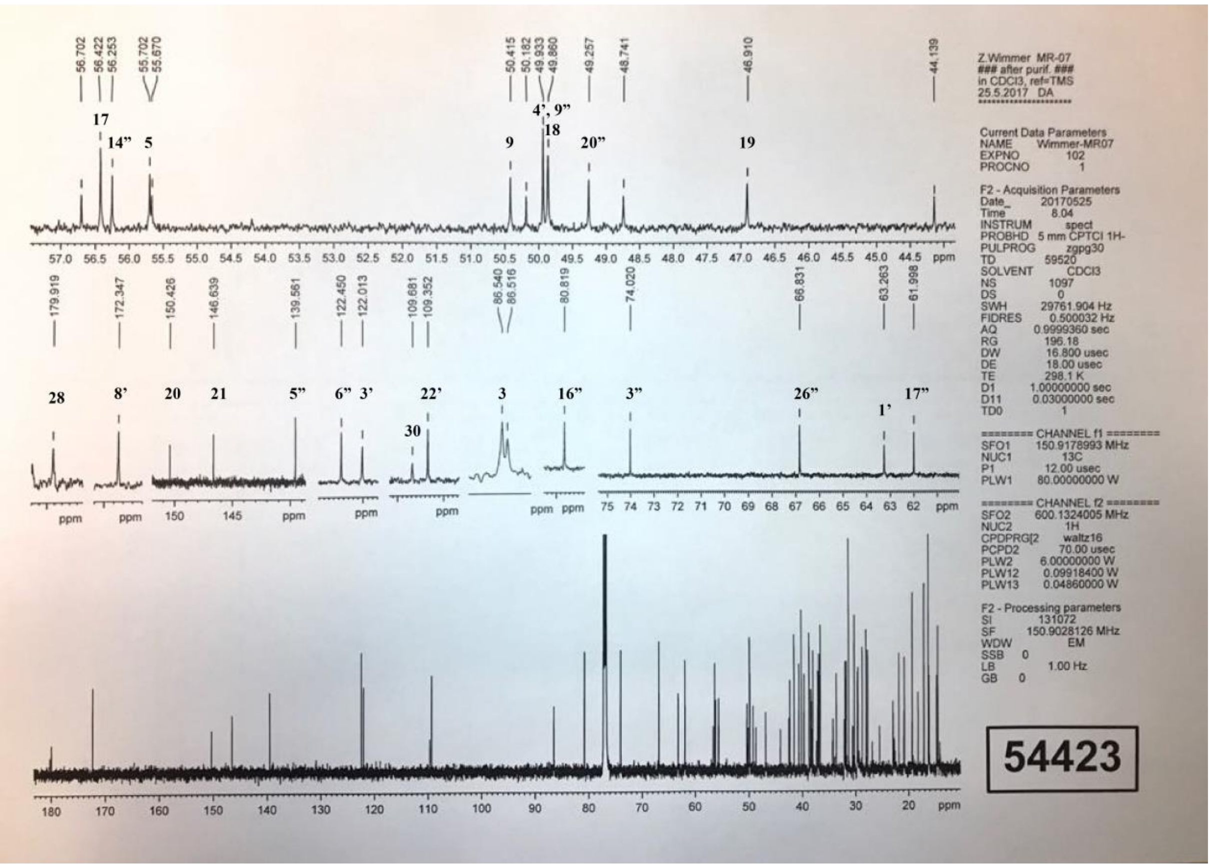
56.702
56.422
56.253
55.702
55.670
50.415
50.182
49.933
49.860
49.257
48.741
46.910
44.139
17
14” 5
4’, 9”
9 18 20”
19
57.0 56.5 56.0 55.5 55.0 54.5 54.0 53.5 53.0 52.5 52.0 51.5 51.0 50.5 50.0 49.5 49.0 48.5 48.0 47.5 47.0 46.5 46.0 45.5 45.0 44.5 ppm
179.919
172.347
150.426
146.639
139.561
122.450
122.013
109.681
109.352
86.540
86.516
80.819
74.020
66.831
63.263
61.998
28
8’
20
21
5”
6” 3’
22’
30
3
16”
3”
26”
1’
17”
ppm ppm 150 145 ppm ppm ppm ppm ppm 75 74 73 72 71 70 69 68 67 66 65 64 63 62 ppm
180 170 160 150 140 130 120 110 100 90 80 70 60 50 40 30 20 ppm
Z.Wimmer MR-07
after purif.
in CDCl3, ref=TMS
25.5.2017 DA
Current Data Parameters
NAME Wimmer-MR07
EXPNO 102
PROCNO 1
F2 - Acquisition Parameters
Date_ 20170525
Time 8.04
INSTRUM spect
PROBHD 5 mm CPTCI 1H-
PULPROG zgpg30
TD 59520
SOLVENT CDCl3
NS 1097
DS 0
SWH 29761.904 Hz
FIDRES 0.500032 Hz
AQ 0.9999360 sec
RG 196.18
DW 16.800 usec
DE 18.00 usec
TE 298.1 K
D1 1.00000000 sec
D11 0.03000000 sec
TD0 1
======== CHANNEL f1 ========
SFO1 150.9178993 MHz
NUC1 13C
P1 12.00 usec
PLW1 80.00000000 W
======== CHANNEL f2 ========
SFO2 600.1324005 MHz
NUC2 1H
CPDPRG[2 waltz16
PCPD2 70.00 usec
PLW2 6.00000000 W
PLW12 0.09918400 W
PLW13 0.04860000 W
F2 - Processing parameters
SI 131072
SF 150.9028126 MHz
WDW EM
SSB 0
LB 1.00 Hz
GB 0
54423

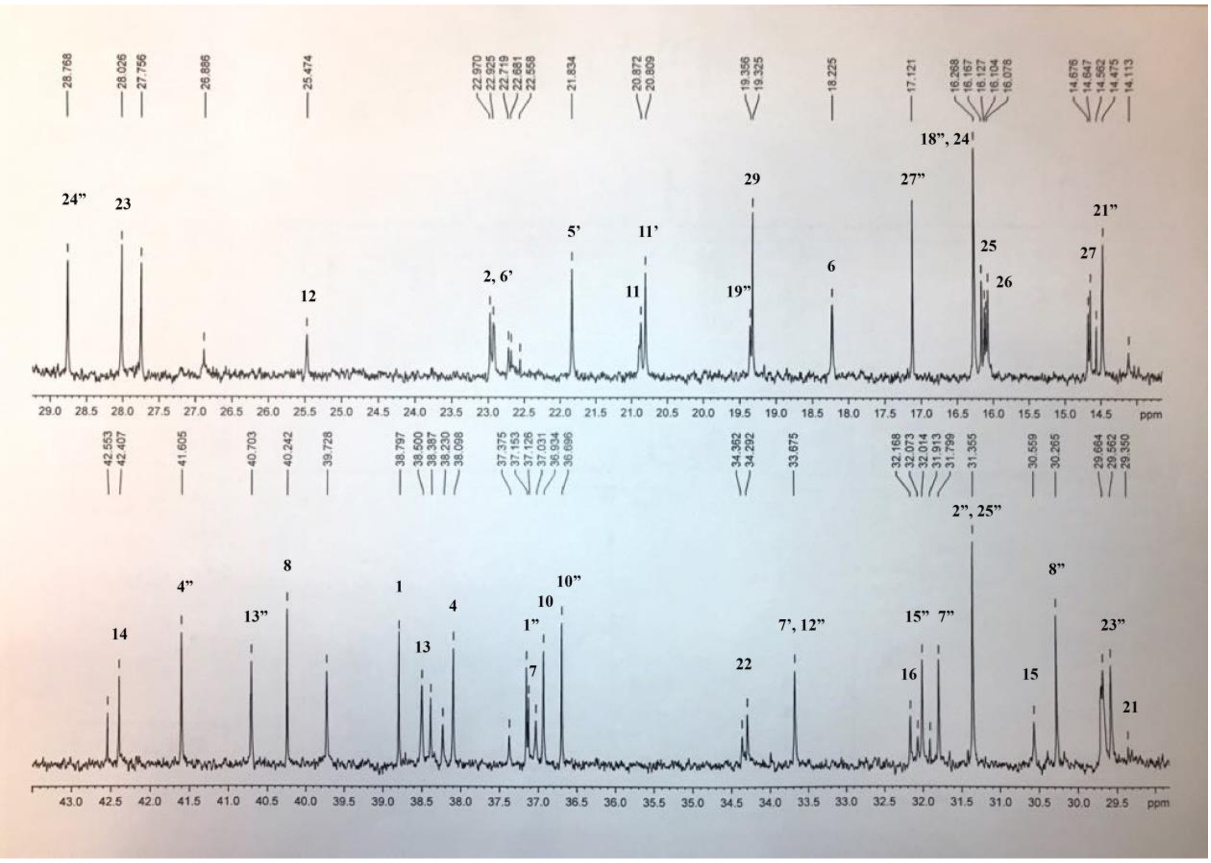
24”
23
12
2, 6’
5’
11’
11
29
19”
6
27”
18”, 24
25
26
27
21”
14
4”
13”
8
1
13
4
1”
7
10
10”
22
7’, 12”
16
15”
7”
2”, 25”
15
8”
23”
21
ppm

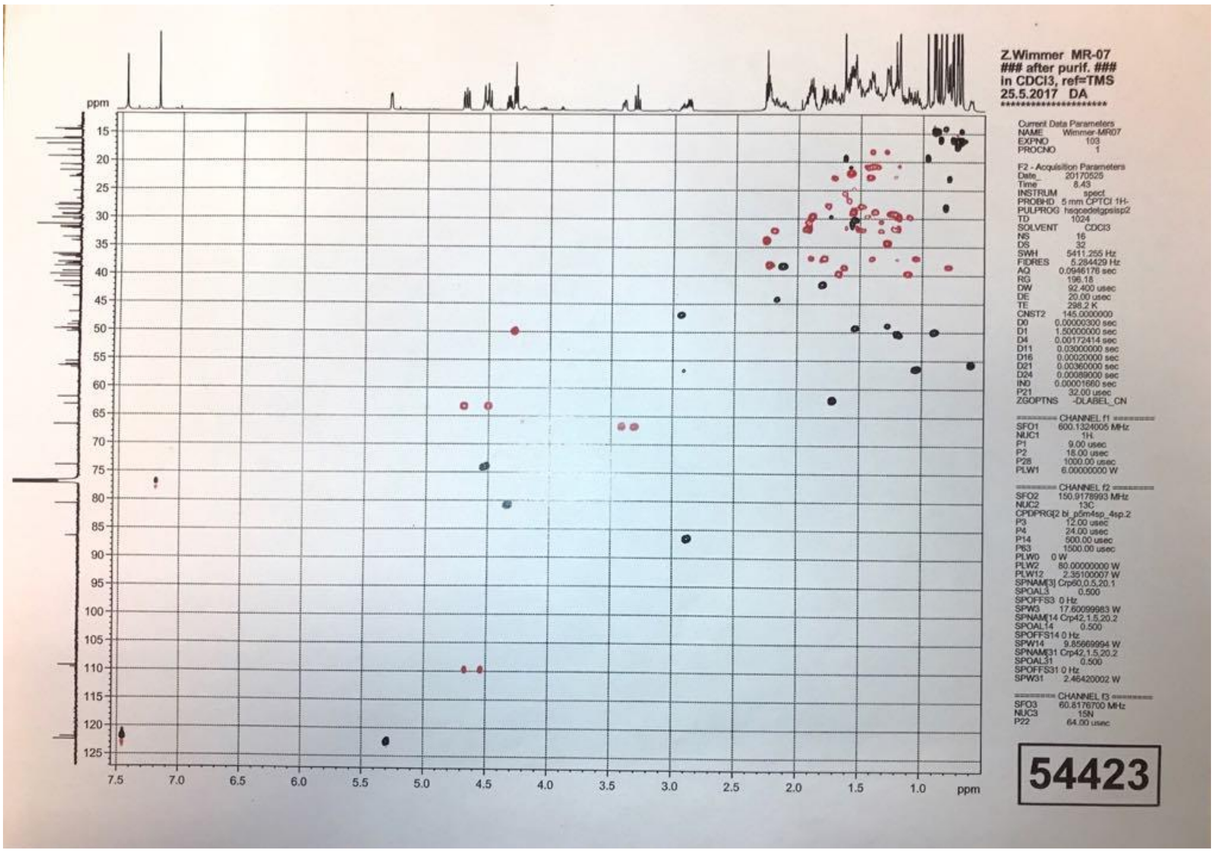
Z.Wimmer MR-07
after purif.
in CDCl3, ref=TMS
25.5.2017 DA
ppm
54423

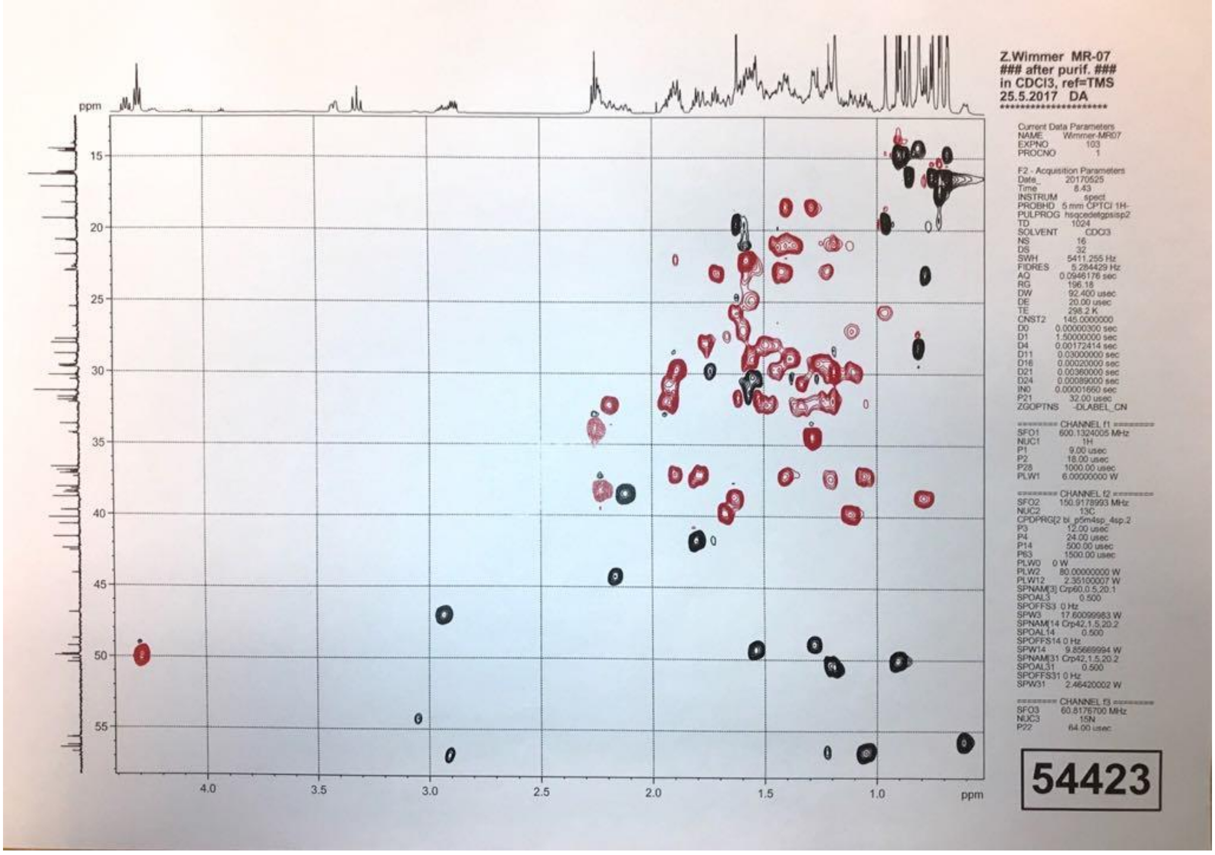

Z.Wimmer MR-07
after purif.
in CDCl3, ref=TMS
25.5.2017 DA
Current Data Parameters
NAME Wimmer-MR07
EXPNO 103
PROCNO 1
F2 - Acquisition Parameters
Date_ 20170525
Time 8.43
INSTRUM spect
PROBHD 5 mm CPTCI 1H-
PULPROG hsqcedetgpsisp2
TD 1024
SOLVENT CDCl3
NS 16
DS 32
SWH 5411.255 Hz
FIDRES 5.284429 Hz
AQ 0.0946176 sec
RG 196.18
DW 92.400 usec
DE 20.00 usec
TE 298.2 K
CNST2 145.0000000
D0 0.00000300 sec
D1 1.50000000 sec
D4 0.00172414 sec
D11 0.03000000 sec
D16 0.00020000 sec
D21 0.00360000 sec
D24 0.00089000 sec
IN0 0.00001660 sec
P21 32.00 usec
ZGOPTNS -DLABEL_CN
======== CHANNEL f1 ========
SFO1 600.1324005 MHz
NUC1 1H
P1 9.00 usec
P2 18.00 usec
P28 1000.00 usec
PLW1 6.00000000 W
======== CHANNEL f2 ========
SFO2 150.9178993 MHz
NUC2 13C
CPDPRG[2 bi_p5m4sp_4sp.2
P3 12.00 usec
P4 24.00 usec
P14 500.00 usec
P63 1500.00 usec
PLW0 0 W
PLW2 80.00000000 W
PLW12 2.35100007 W
SPNAM[3] Crp60,0.5,20.1
SPOAL3 0.500
SPOFFS3 0 Hz
SPW3 17.60099983 W
SPNAM[14 Crp42,1.5,20.2
SPOAL14 0.500
SPOFFS14 0 Hz
SPW14 9.85669994 W
SPNAM[31 Crp42,1.5,20.2
SPOAL31 0.500
SPOFFS31 0 Hz
SPW31 2.46420002 W
======== CHANNEL f3 ========
SFO3 60.8176700 MHz
NUC3 15N
P22 64.00 usec
54423
ppm
4.0
3.5
3.0
2.5
2.0
1.5
1.0
15
20
25
30
35
40
45
50
55
